# Robotic right ventricle is a biohybrid platform that simulates right ventricular function in (patho)physiological conditions and intervention

**DOI:** 10.1038/s44161-023-00387-8

**Published:** 2023-12-08

**Authors:** Manisha Singh, Jean Bonnemain, Caglar Ozturk, Brian Ayers, Mossab Y. Saeed, Diego Quevedo-Moreno, Meagan Rowlett, Clara Park, Yiling Fan, Christopher T. Nguyen, Ellen T. Roche

**Affiliations:** 1 Institute for Medical Engineering and Science, Massachusetts Institute of Technology, Cambridge, MA, USA; 2 Department of Adult Intensive Care Medicine, Lausanne University Hospital and University of Lausanne, Lausanne, Switzerland; 3 Department of Surgery, Massachusetts General Hospital, Boston, MA, USA; 4 Department of Cardiac Surgery, Boston Children’s Hospital, Harvard Medical School, Boston, MA, USA; 5 Department of Mechanical Engineering, Massachusetts Institute of Technology, Cambridge, MA, USA; 6 Department of Biological Engineering, Massachusetts Institute of Technology, Cambridge, MA, USA; 7 Athinoula A. Martinos Center for Biomedical Imaging, Massachusetts General Hospital, Charlestown, MA, USA; 8 Cardiovascular Research Center, Massachusetts General Hospital, Charlestown, MA, USA; 9 Cardiovascular Innovation Research Center, Heart Vascular Thoracic Institute, Cleveland Clinic, Cleveland, OH, USA; 10 Imaging Sciences, Imaging Institute, Cleveland Clinic, Cleveland, OH, USA; 11 Department of Biomedical Engineering, Lerner Research Institute, Cleveland Clinic, Cleveland, OH, USA

## Abstract

The increasing recognition of the right ventricle (RV) necessitates the development of RV-focused interventions, devices and testbeds. In this study, we developed a soft robotic model of the right heart that accurately mimics RV biomechanics and hemodynamics, including free wall, septal and valve motion. This model uses a biohybrid approach, combining a chemically treated endocardial scaffold with a soft robotic synthetic myocardium. When connected to a circulatory flow loop, the robotic right ventricle (RRV) replicates real-time hemodynamic changes in healthy and pathological conditions, including volume overload, RV systolic failure and pressure overload. The RRV also mimics clinical markers of RV dysfunction and is validated using an in vivo porcine model. Additionally, the RRV recreates chordae tension, simulating papillary muscle motion, and shows the potential for tricuspid valve repair and replacement in vitro. This work aims to provide a platform for developing tools for research and treatment for RV pathophysiology.

Etiologies for right ventricle (RV) dysfunction include pressure overload from pulmonary hypertension, volume overload from valvular pathologies and RV systolic failure from cardiomyopathy or myocardial infarction^1–4^. An increasing focus on understanding the role of the right heart in various cardiovascular diseases, along with the growing prevalence of RV dysfunction, has spurred innovation in right heart interventions^2,5^. For instance, the market size for transcatheter tricuspid valve (TV) repair devices is projected to reach $600 million by 2025 (refs. 6,7). Regulatory requirements for RV-focused devices require rigorous testing, yet animal studies are costly, time consuming and highly variable^8^. Benchtop simulators have utility in cardiovascular research, device testing and procedural training and demonstration, offering an alternative or adjunct to in vivo models while recreating hemodynamics in a controlled and reproducible manner. Despite advancements in the development of simulators for replicating left heart dysfunction, there has been less focus on in vitro simulators that accurately represent the hemodynamics, biomechanics and pathologies of the RV^9–11^.

Here we introduce a cardiovascular simulator combining a biorobotic RV and a mock circulatory loop to replicate physiology and function in vitro. The robotic right ventricle (RRV) is the primary pump, recreating the corresponding hemodynamics and obviating the need for an external pulsatile pump, and comprises organic endocardial tissue and a synthetic soft robotic myocardium. By optimizing the biomechanics and hemodynamics of the RRV through computational design and validating the simulator through an in vivo porcine study, we successfully replicated right heart flow, volumes and pressures on the bench and compared functional parameters to in vivo porcine data. By adjusting circuit parameters and actuation of individual soft robotic actuators, we simulated a diverse range of RV dysfunction, including pulmonary arterial hypertension (PAH), tricuspid valve regurgitation (TR) and RV wall myocardial infarction. Furthermore, we simulated the surgical replacement of a pathological TV and evaluated post-repair hemodynamics, demonstrating the utility of the simulator for device testing, technology development and surgical planning. The RRV can also simulate the contractile motion of papillary muscles, accurately recreating tunable chord tension in both healthy and diseased conditions. Practical utility of the RRV includes improving recognition of RV (dys)function, decreasing the volume of animal testing, establishing a platform for developing tools for disease correction and bridging the gap between benchtop and preclinical testing.

## Results

### Hybrid assembly of the RRV

**Extended Data Fig. 1 |:**
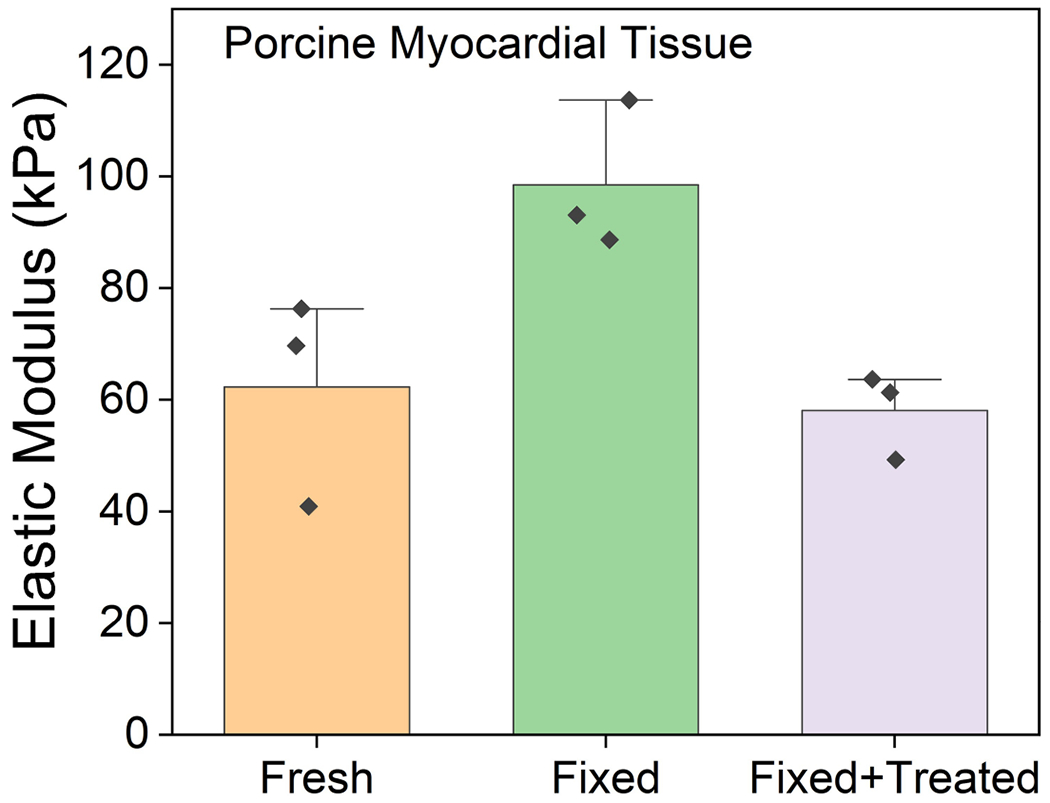
Mechanical properties comparison. Comparison of the elastic mechanical modulus between formalin-fixed and decellularized myocardial muscle tissue samples, demonstrating the impact of the decellularization process on restoring tissue pliability and mechanical properties. Data shows mean ± stdev (n = 3 myocardial samples obtained from n = 1 porcine heart for each type).

**Fig. 1 |:**
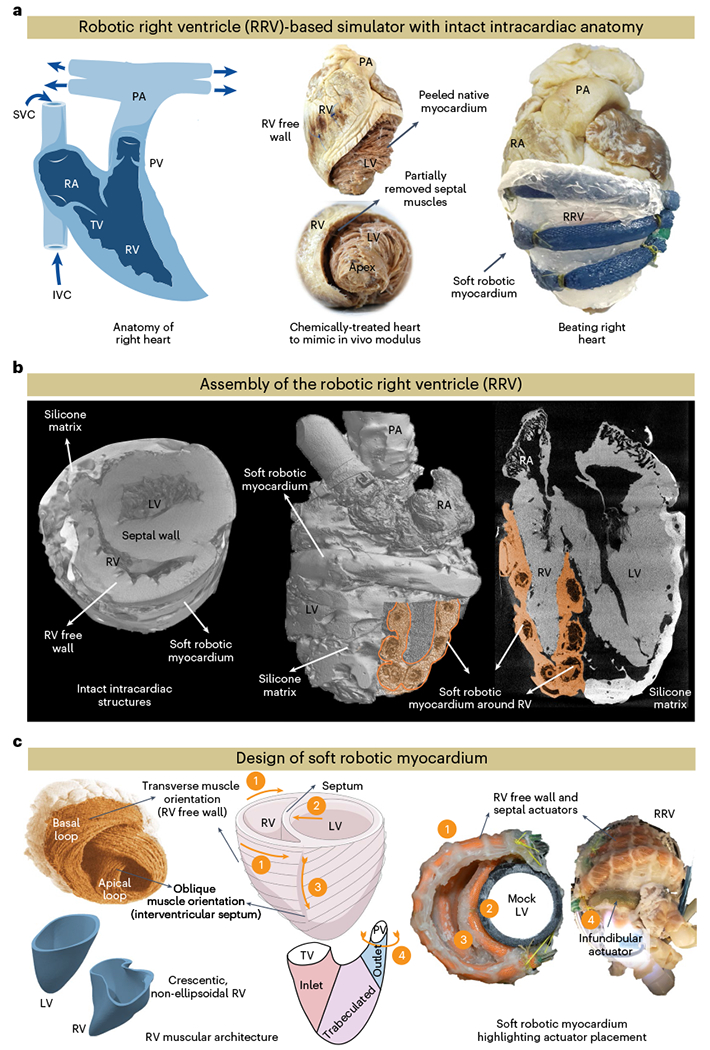
An overview of the RRV and its architecture. **a**, Overview of the bioinspired and biomimetic approach used to create a biohybrid beating right heart. Initially, a freshly explanted porcine heart underwent chemical treatment, after which the native myocardium was hand-dissected and replaced with a robotic counterpart while preserving the endocardial scaffold. **b**, The assembly of the RRV was imaged with micro-CT, revealing the preserved intracardiac structures. **c**, Schematic of the RV’s complex shape, fiber orientation and wall motion (left). The image of the heart showing fiber orientation in the basal and apical loops is reprinted from Buckberg et al.^12^, with permission from Elsevier. The physical model of the soft robotic myocardium shows the placement of individual actuators in the synthetic myocardium (right). The outflow tract of the RV contains infundibular muscles that also contribute to RV ejection through contraction. The contraction of the outflow region can be achieved by placing a circumferential actuator across the infundibulum. The numbers denote the respective actuator placement corresponding to the RV motion shown in the schematics on the left. SVC, superior vena cava.

The RV features complex three-dimensional (3D) endocardial structures, including valves, trabeculae, papillary muscles and the moderator band. Traditional manufacturing methods limit recreating these structures accurately due to a lack of elastic materials suitable for high-precision 3D printing. We developed RRV using a biohybrid approach to overcome these limitations in additive manufacturing. To achieve anatomical accuracy, we initially preserved fresh pig hearts with formalin and then treated them with surfactants to restore native tissue-like properties (Extended Data Fig. 1). We then dissected the thick myocardial tissue from the left ventricle and interventricular septal area, following the natural tissue fiber orientation. The removed tissue was replaced with a synthetic elastomeric myocardium featuring McKibben-style soft robotic actuators aligned with the underlying muscle architecture in a biomimetic manner, replicating the contractile function of native myocardium to achieve physiologically accurate RV motion (Fig. 1a). These actuators, with an input pressure of 25 psi, can generate a 25% axial contraction and a 117% radial expansion, producing sufficient intraventricular pressure for physiologically relevant volume displacement (Supplementary Fig. 1). Imaging the RRV with computed tomography (CT) provides a 3D view of the architecture and anatomy of the RRV (Fig. 1b), revealing the preserved anatomical details and the integration of soft robotic actuators into the RV free wall and septum.

### Computational design optimization of the soft robotic muscle

The RV contracts in a different manner than the left ventricle (LV)^2^. RV contraction involves four mechanisms (Fig. 1c, left): (1) The RV free wall moving inward, (2) the septum bulging into the RV, (3) longitudinal contraction moving the TV annulus toward the apex and (4) circumferential contraction of the right ventricular outflow tract (RVOT). The ventricles have a 3D array of fibers forming basal and apical loops^12,13^. The basal loop, with transverse muscle orientation predominantly moves the RV free wall. The apical loop, with oblique muscle orientation, contributes to the RV/LV septum and LV lateral wall motion. The LV’s torsional movement is passively transferred to the RV, resulting in a peristaltic contraction^2^. Our soft robotic myocardium replicates the natural fiber orientation of cardiac muscles, and its actuators mimic these mechanisms, with placements corresponding to these numbers (Fig. 1c, right): a circumferential actuator induces RV outflow region contraction while actuators along the RV trabeculated region and intraventricular septum mimic RV free wall and septal wall motion.

**Fig. 2 |:**
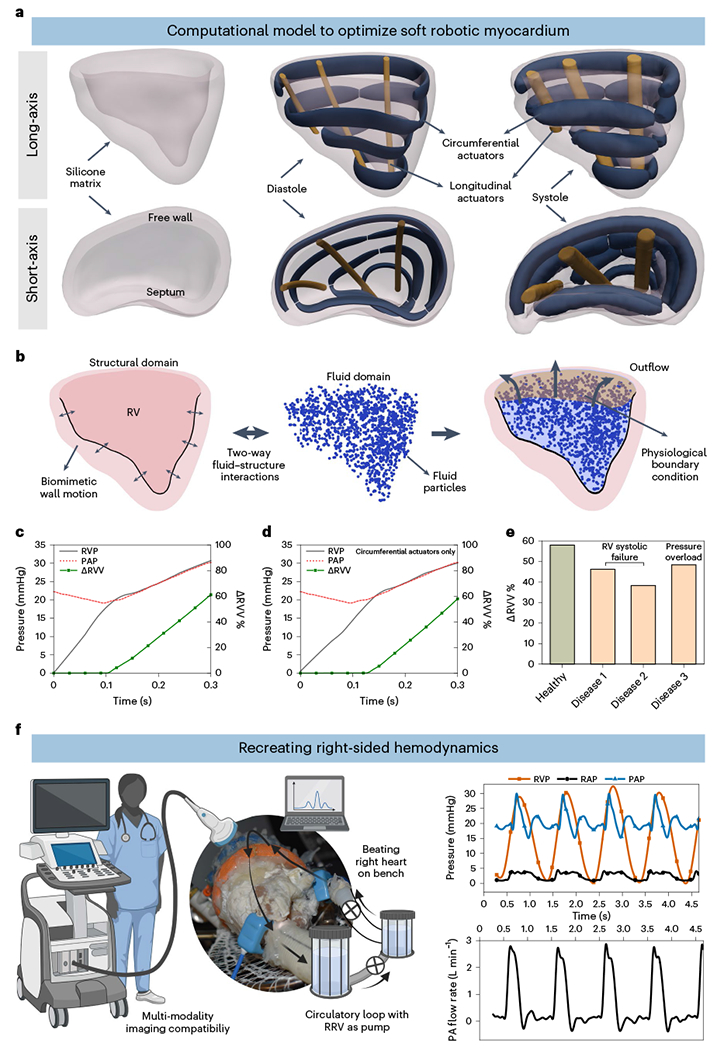
Computational and in vitro modeling of the RV biomechanics. **a**, FE model of the soft robotic myocardium, highlighting the placement and spatial density of the circumferential and longitudinal actuators. The computational myocardium exhibits structural deformations during both diastolic and systolic phases, which can be observed from long-axis (top) and short-axis (bottom) views. **b**, Schematics illustrating the design principles of modeling two-way fluid–structure interaction. **c**, Prediction of ejected chamber volume (ΔRVV) and RVP during systole under physiological boundary conditions for both longitudinal and circumferential actuators. **d**, Prediction of ΔRVV and RVP during systole under physiological boundary conditions for circumferential actuators (no longitudinal actuators). **e**, Prediction of ΔRVV for various disease cases by selectively deactivating actuators and increasing the hemodynamic after-load. Disease cases 1 and 2 represent varying degrees of RV systolic failure, whereas disease case 3 recapitulates a pressure overload condition. **f**, Physiological hemodynamics are replicated in the RRV cardiovascular simulator, which produces pressure and flow waveforms that accurately represent those of the human heart.

To optimize the motion and function of the biomimetic soft robotic myocardium, we developed a computational framework that includes a finite element (FE) model of soft actuators arranged in an idealized RV geometry (Methods). We evaluated the global behavior of the soft robotic myocardium using two-way fluid–structure interaction models, coupling FE and computational fluid dynamics (CFD) (Fig. 2a,b and Supplementary Movie 1). The actuator type and spatial density were evaluated based on RV wall motion and pressure required for a right ventricular volume reduction (ΔRVV) of 50% or greater. We achieved healthy hemodynamic performance with a peak RV systolic pressure of 32 mmHg and ΔRVV of 62% using a bioinspired design of the robotic myocardium (Fig. 2c). A simplified design with circumferential actuators and no longitudinal actuators resulted in similar hemodynamics, with a peak RV pressure of 30 mmHg and a ΔRVV of 58% (Fig. 2d). We used this simplified design for the fabrication of the RRV.

**Extended Data Fig. 2 |:**
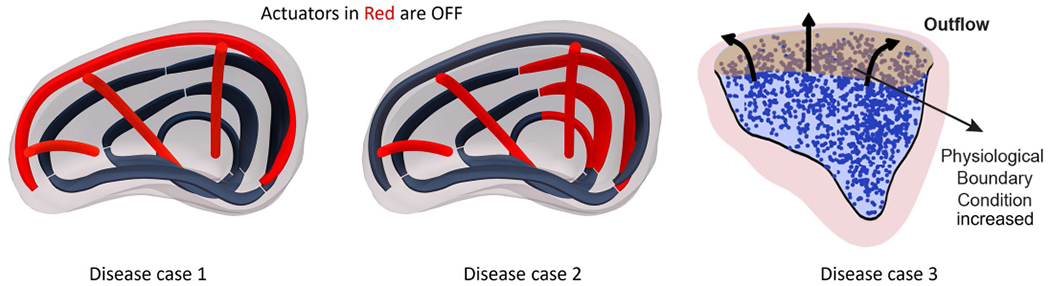
Computationally recreated pathology. Disease cases 1 and 2 represent systolic right ventricular failure and were computationally recreated by deactivating the actuators colored in red. Disease case 3 was created by increasing the time-dependent pressure boundary condition to 40 mmHg during systole.

The ability of the synthetic myocardium to recreate RV pathology was explored by selectively deactivating individual soft robotic elements and by increasing the systolic outflow pressure boundary conditions to 40 mmHg. We measured the impact on ΔRVV, with varying degrees of deactivation. For example, we simulated RV systolic dysfunction, in cases 1 and 2, by switching off the top (basal) and bottom anterior wall actuators, respectively (Extended Data Fig. 2). Furthermore, we recapitulated pressure overload (disease case 3) by increasing the physiological pressure boundary condition at the pulmonary outflow to 40 mmHg. The effect of each disease case on the ΔRVV is shown in Fig. 2e. The clinically defined normal range for ΔRVV, representing ventricular ejection, is greater than 45%. This computational strategy allowed us to explore parameters such as spatial density, placement of actuators and resulting hemodynamics before experimental characterization.

### Recreating healthy right-sided hemodynamics

**Extended Data Fig. 3 |:**
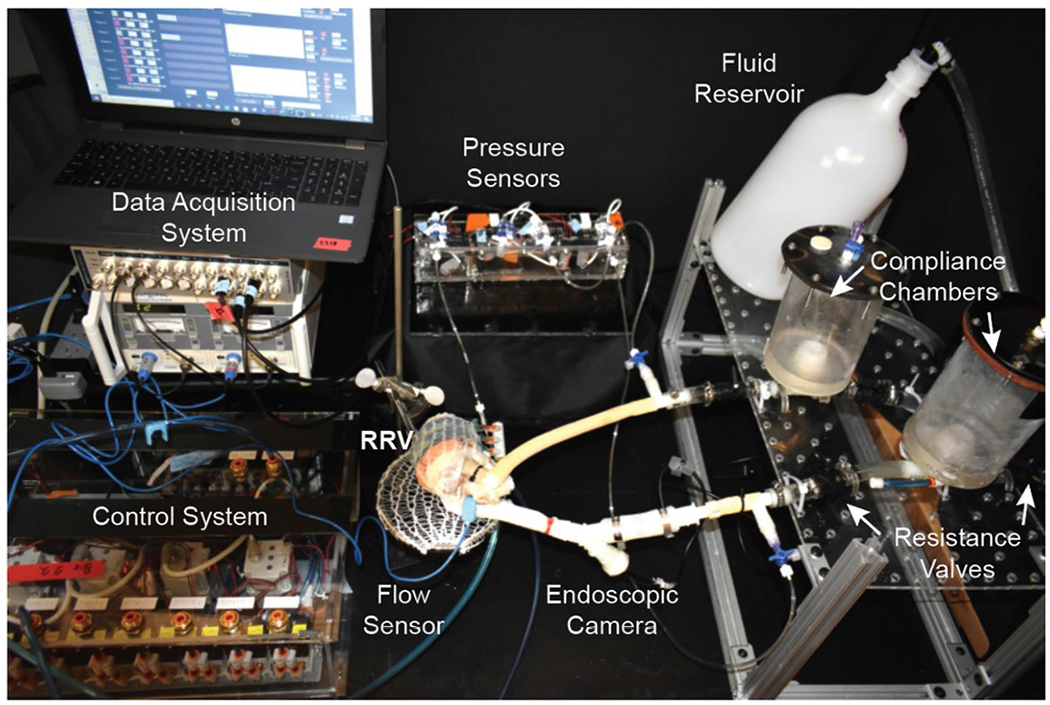
Experimental fluid model. The setup of the mock circulatory flow loop.

We designed a mock circulatory flow loop that simulates pulmonary circulation and allows for the adjustment of parameters such as pre-load, after-load, vascular compliance and resistance (Extended Data Fig. 3). The robotic heart serves as the primary pump that drives fluid flow in the circuit, and the system does not require an additional pump (Supplementary Movie 2). The actuators were pressurized using a control system, and a sequential delay of 20–50 ms was applied to simulate peristaltic-like RV ejection. As seen in Fig. 2f, the RRV was able to generate physiological right heart pressures, including phasic right ventricular pressures (RVPs) of 28/3 mmHg, mean right atrial pressures (RAPs) of 3 mmHg and phasic pulmonary artery pressures (PAPs) of 25/15 mmHg, with a pulmonary artery (PA) outflow of over 3 L min^−1^ at a heart rate of 60 beats per minute (bpm).

### Validation with in vivo porcine models

**Fig. 3 |:**
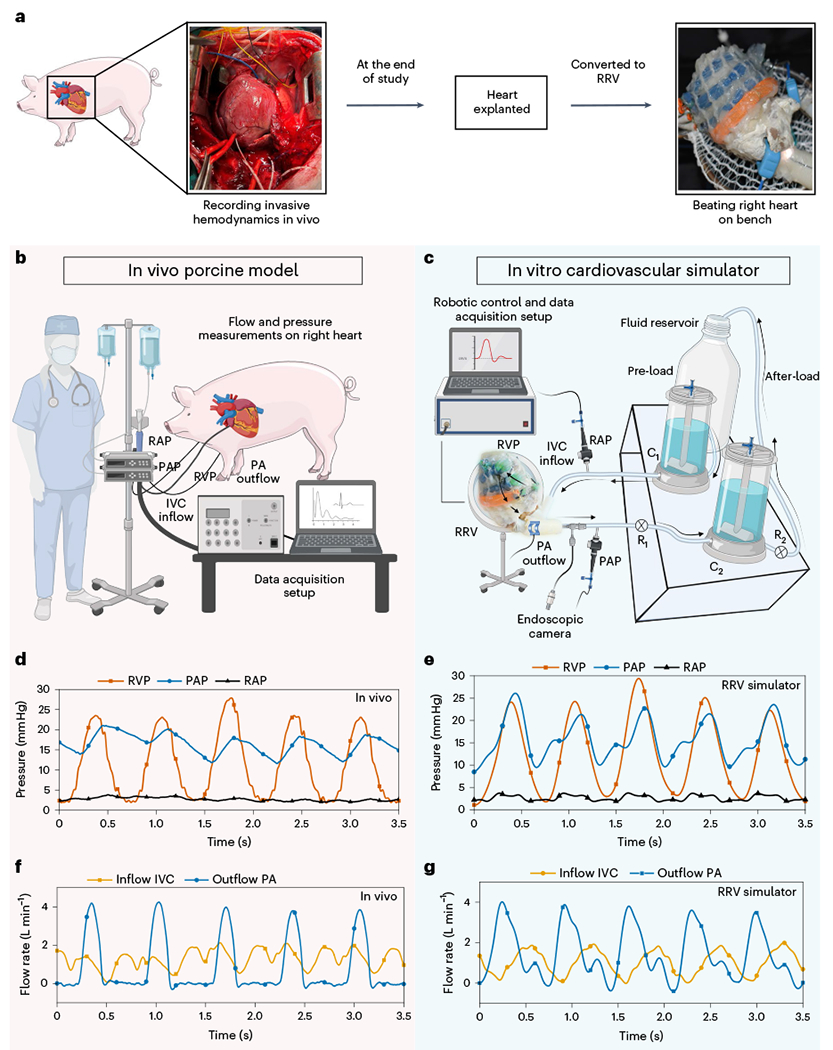
Comparison of in vivo and in vitro RRV hemodynamics. **a**, The RRV was constructed using a freshly explanted porcine heart. Hemodynamic data were collected from juvenile pigs in vivo and used to validate the accuracy of the in vitro model. **b**, The surgical and data acquisition setup to record invasive hemodynamic measurements in pig models. **c**, A mock circulatory flow loop incorporating hydraulic and mechanical components to simulate the pulmonary and systemic circulation on a laboratory bench. The RRV serves as the primary pump, driving flow through the loop. **d**, Right-sided pressure waveforms recorded in vivo. **e**, In vitro right-sided pressure waveforms were recreated using the RRV and the mock circulatory flow loop. **f**, In vivo flow waveforms show inflow and outflow. **g**, Representative flow waveforms recapitulated in vitro.

To validate the accuracy of our benchtop RRV simulator in generating physiological function, we compared the hemodynamic parameters obtained from healthy juvenile pigs to those recreated in our in vitro system (Fig. 3a–c). The results show good agreement between the two systems. Representative examples of RAP, RVP and PAP for both in vivo and in vitro models are displayed for comparison (Fig. 3d,e). The pressure magnitudes and waveform shapes match closely in both cases, with a systolic RVP of 25 ± 4 mmHg, stable mean RAP of 4 ± 2 mmHg and PAP of 13 ± 4 mmHg in diastole. The pressure differences between the chambers of the right heart demonstrate that both the TV and pulmonary valve (PV) are competent under physiological pressures. Similarly, the flow waveforms for inferior vena cava (IVC) inflow and PA outflow recapitulate the in vivo data. The quantitative flow patterns between inflow and outflow suggest that the flow driven by the soft robotic myocardium is unidirectional and effectively regulated by the tissue valves in the endocardial scaffold (Fig. 3f,g). The baseline fluctuations in IVC flow and PA pressures in the in vivo model are a result of the impact of respiration on right heart circulation and are not present in the in vitro model.

**Extended Data Fig. 4 |:**
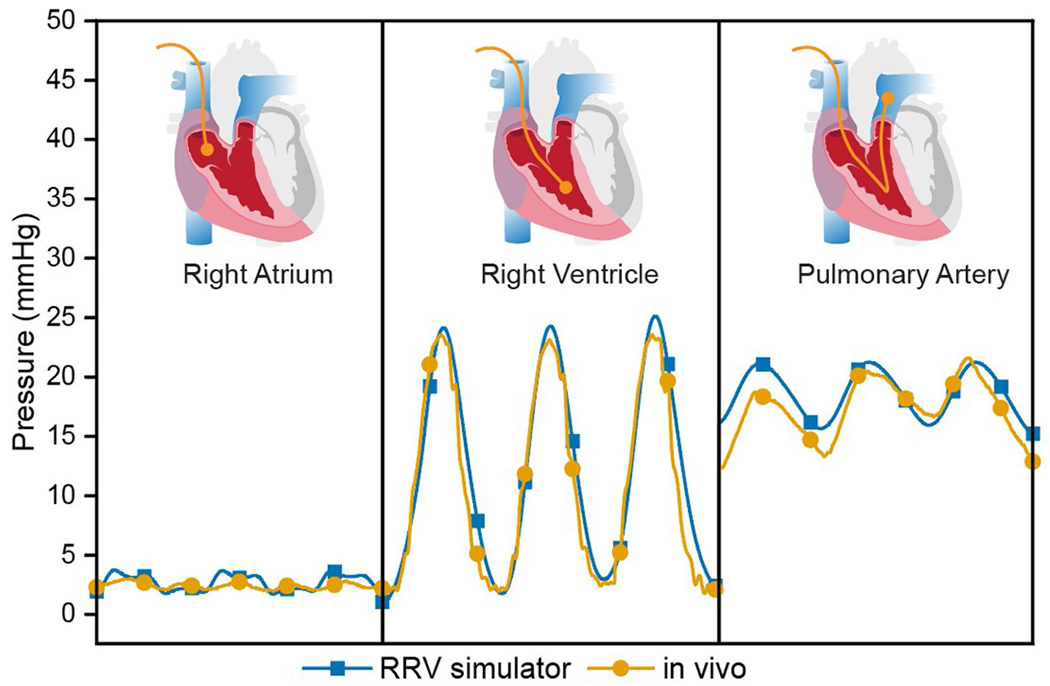
Hemodynamic parameter tunability. Tunability of the pulmonary artery pressure (PAP) waveform in the simulator can be achieved by adjusting circuit parameters to simulate in vivo overdamping. This results in a PA pressure waveform that more closely matches the overdamped signal observed in vivo.

**Fig. 4 |:**
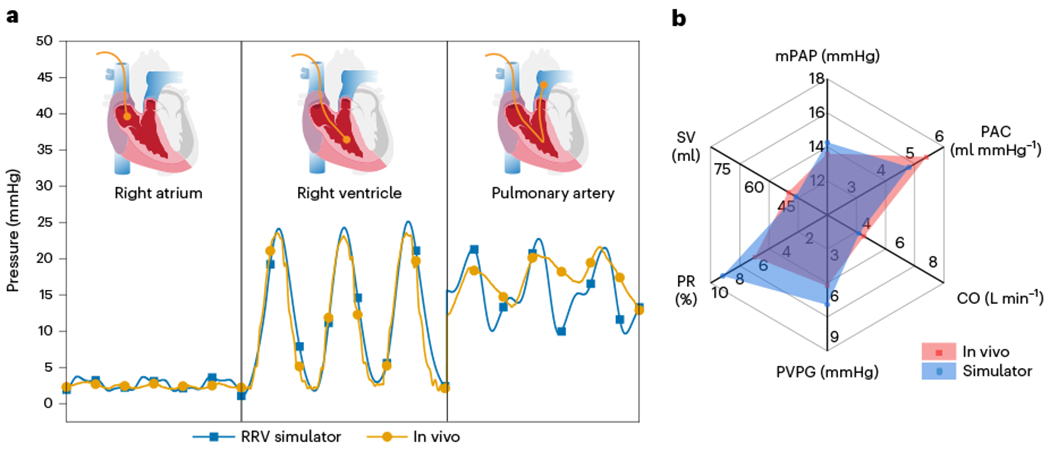
A comparison of in vivo and in vitro pressure waveforms. **a**, In vivo and in vitro pressure waveforms from the RA, RV and PA. **b**, The similarities between clinical indicators estimated from in vivo pig and in vitro right heart simulator hemodynamic data are highlighted using a web spider plot. Parameters include cardiac output (CO), mean pulmonary artery pressure (mPAP), pulmonary arterial compliance (PAC), stroke volume (SV), pulmonary transvalvular pressure gradient (PVPG) and pulmonary regurgitation (PR).

Figure 4a shows a comparison of the overlaid RVP, PAP and RAP, demonstrating the close match between the animal and benchtop data. The in vivo PA pressures display overdamping, which suggests an overestimation of diastolic blood pressure and waveform attenuation. These artifacts can be difficult to manage in animal studies but can be adjusted in a benchtop simulator. The PA pressure waveform generated on the bench closely resembles textbook waveforms, with the downslope of the diastolic portion and a dicrotic notch. By fine-tuning the resistance R1 and R2 and compliance C2 in the afterload circuit, we can easily replicate the in vivo overdamping of PAP, as shown in Extended Data Fig. 4. The results validate the robustness and accuracy of our benchtop right heart model in generating physiological function and its potential for use in pre-clinical and clinical applications. Extended Data Table 1 outlines the distinctive features and advantages of the RRV when compared to existing in vitro and ex vivo models.

We measured clinical parameters of the right heart and cardiopulmonary physiology, including cardiac output (CO), mean pulmonary artery pressure (mPAP), pulmonary arterial compliance (PAC), stroke volume (SV), pulmonary transvalvular pressure gradient (PVPG) and pulmonary regurgitation (PR). The similarity between RV functional parameters obtained in in vivo and in vitro studies is shown in Fig. 4b. The clinical determinants for RRV were within the physiological range, with precise values of 14.26 mmHg for mPAP, 4.8 ml mmHg^−1^ for PAC, 3.9 L min^−1^ for CO, 6.6 mmHg for PVPG, 8.95% for PR and 44.83 ml for SV. Finally, the RVPs and the volumes of the RV, which were calculated using a combination of inflow, outflow and anatomy data, were analyzed to compare the pressure–volume relationship in vitro and in vivo. The pressure–volume loops of the RV in the in vitro study were trapezoidal or triangular in shape, which is consistent with the findings reported in the literature for human in vivo studies, as shown in Supplementary Fig. 2, differing from the characteristic cylindrical shape described in the literature for the LV pressure–volume loops.

### Mimicry of right-sided anatomical and functional features

**Fig. 5 |:**
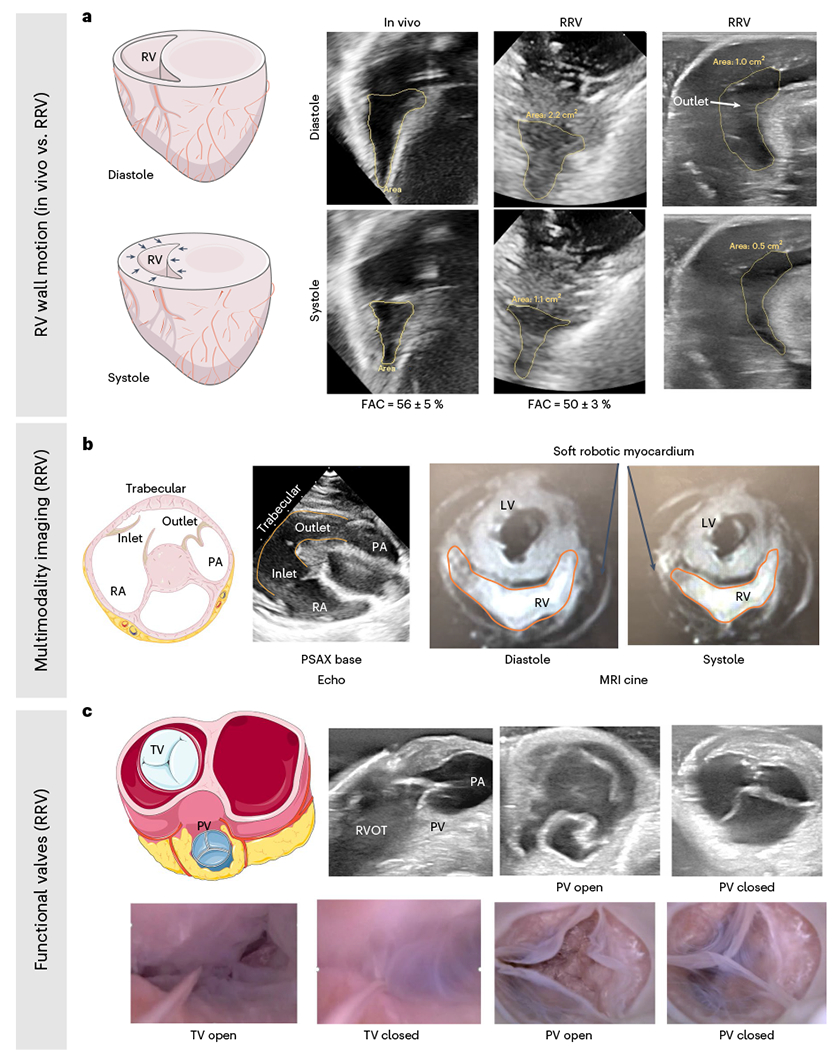
Imaging the motion and valve function of the RRV. **a**, The motion of the right ventricular free wall and intraventricular septum in the RRV is compared to in vivo porcine data, revealing similarities in the FAC (in vivo 56 ± 5% versus in vitro 50 ± 3%). **b**, The RRV model can be imaged using multiple modalities. An echocardiography image of the parasternal short-axis base view (left), which displays the inflow, outflow and trabeculated regions of the RV, and an MRI cine image (right), depicting the RV chamber’s circumferential contraction during diastole and systole, are used to demonstrate the RV’s anatomy and function. **c**, The functional PV and TV in the RRV are visualized using echocardiography (top) and an endoscopic camera (bottom).

The bioinspired design of the soft robotic myocardial substitute enables mimicry of the movement of the RV wall. We employed both transthoracic and epicardial echocardiography techniques to obtain images of the heart. Subsequently, we transformed the pig heart into the robotic (RRV) configuration and actuated it while conducting epicardial echocardiography. In Fig. 5a, the echocardiogram images showcase the clear movement of the right ventricular free wall and septum, made possible by the inflation of the soft robotic actuators during systole and their deflation during diastole. The fractional area change (FAC) of the right ventricular chamber was similar in the RRV and in vivo. The FAC is 50 ± 3% and 56 ± 5% in the RRV and in vivo, respectively. Healthy values of FAC should be greater than 38% in swine and greater than 42% in humans^14^. By incorporating an additional actuator across the infundibular region of the muscle, we were able to produce circumferential contraction of the RVOT during systole, as shown in the right-most echocardiographic images (Fig. 5a).

The RRV platform is compatible with a variety of imaging techniques, including ultrasound, CT scans and magnetic resonance imaging (MRI). Figure 5b displays a conventional parasternal short-axis view obtained through echocardiography. This view, known as parasternal short axis (PSAX) base, enables two-dimensional (2D) evaluation of the TV, PV, right ventricular function and collapse of the right atrium (RA) during systole^15–17^. A frame from the MRI cine taken at the end of diastole and systole is shown in Fig. 5b and Supplementary Movie 3, which highlights the contraction of the RV during systole. The soft robotic myocardium surrounding the endocardial structures can also be seen under MRI.

The soft robotic myocardium generates a peristaltic-like contraction, propelling fluid forward and causing pressure differentials across the TV and PV, which results in their timed opening and closing. The 2D views of the valves can be visualized through echocardiography and endoscopic cameras (Fig. 5c and Supplementary Movie 3). During diastole, the TV opens for right ventricular filling, and, during systole, it closes tightly to prevent backflow to the RA. The physiological motion of the right ventricular wall mimics the biomimetic motion of the TV. The shortening of the RV chamber approximates the papillary muscles and causes the coaptation of TV leaflets. Finally, during systole, the PV opens to eject fluid into the PA and closes during diastole, allowing for RV filling and the development of PA diastolic pressure.

### Intraventricular flow velocity using MRI measurements

**Fig. 6 |:**
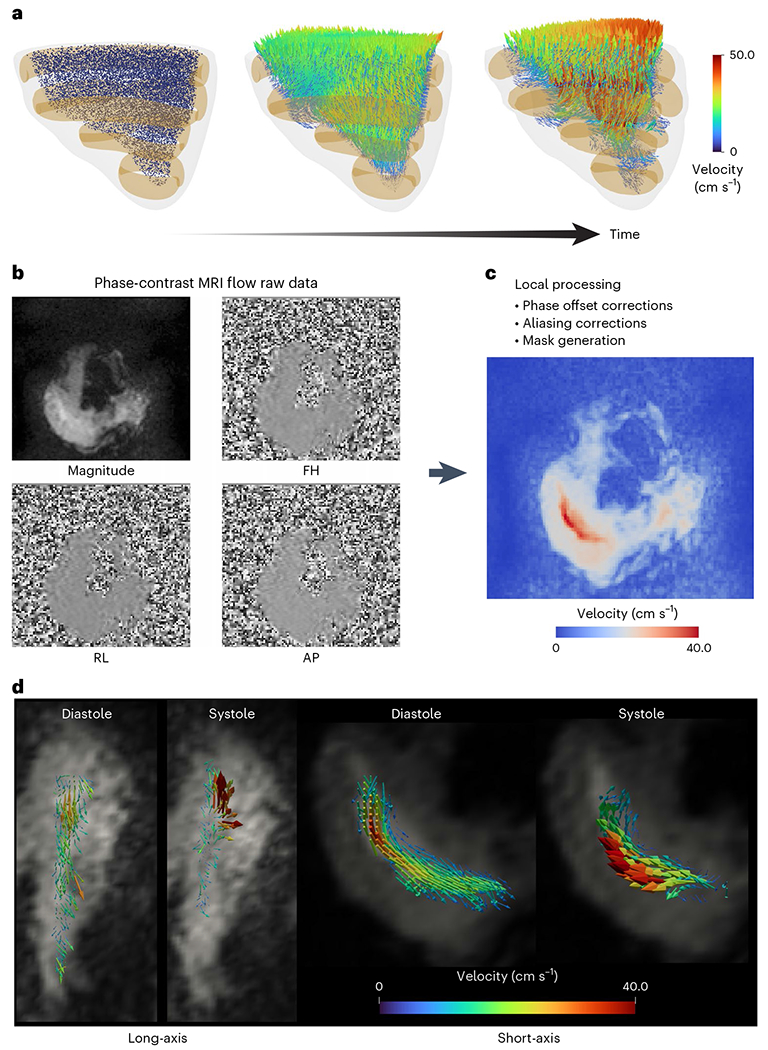
CFD models and MRI velocimetry. **a**, Computational fluid model to predict the interventricular fluid particle velocity. **b**, The magnitude and phase data of a 2D MRI flow measurement sequence are depicted. AP, anterior-posterior direction; FH, foot–head; RL, right–left. **c**, The cross-sectional data recorded during the study were carefully analyzed and corrected to accurately depict the distribution of flow across the RV. **d**, Reconstructed velocity vectors superimposed on the long-axis and short-axis cine images during both systole and diastole.

Studying RV dysfunction and flow using MRI in large animals is challenging for several reasons related to animal care, cost and anesthesia. Conventional, rigid benchtop simulators, even if MRI compatible, cannot provide information on intraventricular flow energetics due to their lack of anatomical accuracy. The MRI compatibility and high anatomical and functional accuracy of our RRV facilitates flow measurements inside the RV. Before measuring intraventricular fluid velocity on the benchtop model, we assessed whether our soft robotic myocardium could generate physiologically relevant RV blood velocity using a computational model described in the previous sections (Fig. 6a). Our fluid–particle simulations yielded peak velocity ranges of 40–50 cm s^−1^, which are consistent with human data reported in the literature^18–20^.

We analyzed the MRI phase-contrast 2D flow data to determine the direction and velocity of fluid entering and exiting the right ventricular cavity (Fig. 6b,c). Our analysis revealed a peak fluid velocity of 40 cm s^−1^ across the RV and RVOT. During systole, we observed negligible fluid flow in the trabeculated region of the RV, and fluid predominantly moved from the inlet to the outlet region, as shown in Fig. 6d. The apical region of the RV exhibits minimal involvement during systole with dominant diastolic flow events. These findings corroborate literature on the RV flow pattern^1,2,18–20^.

### Soft robotic muscle can generate RV dysfunction

RV dysfunction is mainly determined by the hemodynamic load (pre-load and after-load), contractility (inotropy) and cardiac frequency^3^. We simulate RV pathologies in the laboratory by deactivating specific actuators, adjusting input pressure and manipulating circulatory system parameters associated with right-sided valvular disease, RV contractility and after-load. As exemplary case studies, we simulate pathological conditions of TR, myocardial infarction and PAH.

**Fig. 7 |:**
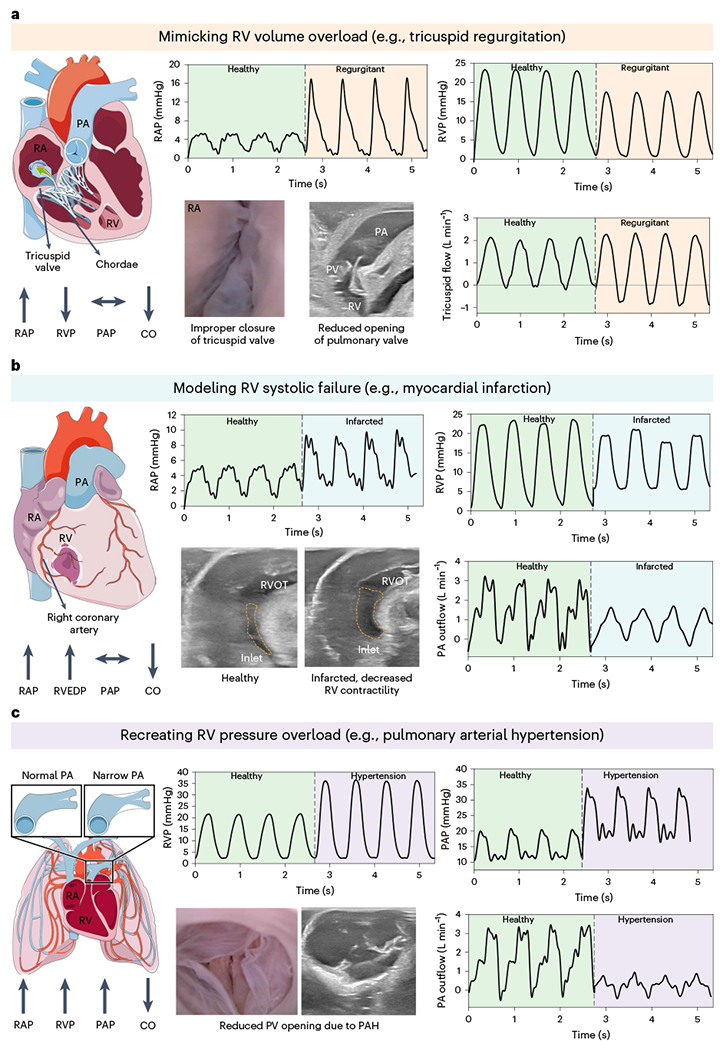
The benchtop RRV can mimic various pathological conditions that affect the right side of the heart. **a**, RV volume overload is illustrated through TR. **b**, RV systolic failure is mimicked through a case example of RVMI. **c**, RV pressure overload is recreated by PAH.

TR occurs when blood flows backward into the RA during the ventricular systole phase, caused by the improper closure of the TV leaflets^21^. We simulated functional TR in our simulator by temporarily deactivating the basal actuator, resulting in partial contraction of the TV annulus and creating a scenario of a dilated annulus. The hemodynamic recordings in Fig. 7a show the impact of TR on the system. The RAP rises to over 16 mmHg, like the systolic RV pressure, indicating substantial leakage through the TV annulus. The flow through the TV was measured using a flow sensor, and the results in Fig. 7a show an increase in backward flow leading to lower systolic RVPs and decreased pressure buildup. Endoscopic images further show inadequate leaflet coaptation of the TV. Echocardiographic views of PV reveal a reduction in the effective orifice area during systole, which suggests that the flow generated by ventricular contraction is divided between the backward and forward directions (Supplementary Movie 4).

**Extended Data Fig. 5 |:**
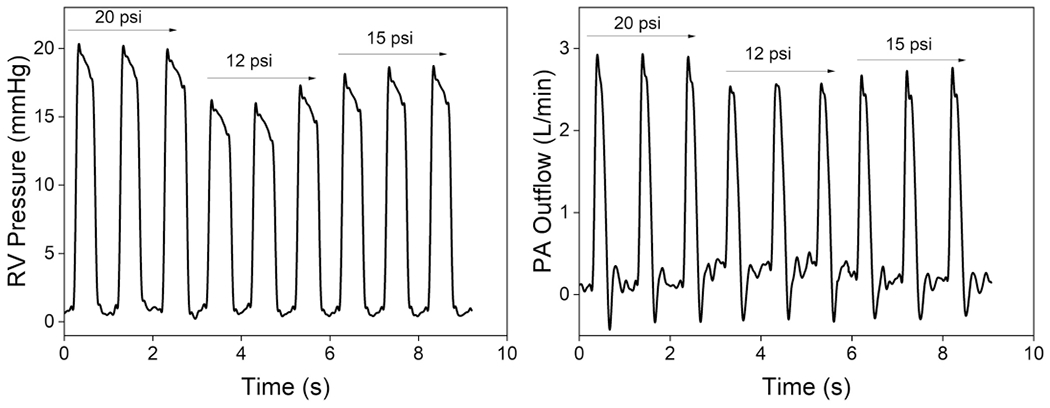
Hemodynamic consequences of RV global dysfunction. Real-time right ventricle (RV) pressure data and pulmonary artery (PA) outflow data at actuation pressures of 12, 15, and 20 psi to replicate the hemodynamic consequences of RV global dysfunction.

Right ventricular myocardial infarction (RVMI) is a condition in which the wall of the RV experiences ischemia due to occlusion of the right coronary artery, resulting in a decline in its contractile function^22^. In cases of RVMI, wall motion anomalies of the RV (hypokinetic or akinetic motion) are predominantly observed below the point of coronary occlusion. In our simulator, we simulate acute RVMI by temporarily deactivating half of the soft robotic components on the inferior wall of the RV, mimicking acute conditions with focal defects such as a proximal or medial occlusion in the right coronary artery. The echocardiographic images in Fig. 7b and Supplementary Movie 4 show a decrease in the shortening of the RV inlet region. The hemodynamic recordings reflect this with an increase in right ventricular end-diastolic pressure (RVEDP) and a decrease in RV contractility, leading to a reduced SV as seen in the PA outflow. Complications associated with RVMI can introduce TR, which is characterized by a 40% increase in mean pressure within the RA. The reduced RV systolic function results in a reduced stroke work index of 0.16 mmHg·L/m^2^ (healthy index is ~0.47 mmHg·L/m^2^). The clinical threshold for RV stroke work index is defined as 0.25 mmHg·L/m^2^, and values below this indicate RV dysfunction^3^. The outcomes demonstrate our model’s ability to replicate the overall consequences, such as reduced CO and elevated RAP and RVEDP, resulting from localized abnormal wall motion, as seen in RVMI scenarios. RV inotropic dysfunction can also manifest globally under certain circumstances, such as RV dysfunction after air emboli during cardiac surgery and after extra-corporeal circulation or in instances of non-ischemic dilated cardiomyopathy. To reproduce comprehensive RV global systolic function, real-time RV pressure data and PA outflow data at actuation pressures of 12 psi and 15 psi are presented in Extended Data Fig. 5. This also holds significance in accurately replicating regional wall abnormalities, which can have broader implications for the global RV function in the acute stage.

PAH is a medical condition marked by elevated mean arterial pressure due to various cardiovascular and respiratory disorders^23^. One contributing factor is increased pulmonary resistance caused by the constriction or obstruction of the PA and smaller arteries in the lungs. In our simulator, we recreate the pathological hemodynamics of this maladaptive remodeling by increasing the after-load resistance R1, similar to pulmonary banding^8,24^. This results in an increase in systolic RV pressure and phasic PA pressure (Fig. 7c). The decrease in PA outflow suggests that RV systolic function is sensitive to changes in after-load, with even minor increases, leading to substantial reductions in SV. PA compliance is a clinical metric used to evaluate the RV–PA coupling in PAH and is considered a marker of RV function with a threshold of 2.5 ml mmHg^−1^ or higher^3^. In our in vitro platform, the PA compliance is 4.2 ml mmHg^−1^ for healthy cases and 0.65 ml mmHg^−1^ for PAH. Abnormal values of PA compliance are a hallmark of RV dysfunction. Coupled with the pressure and flow data, the decreased PA compliance and reduced CO also indicate the successful recreation of PAH in our robotic heart simulator (Supplementary Movie 4).

### RRV as a testbed for surgical training and device evaluation TV replacement.

**Fig. 8 |:**
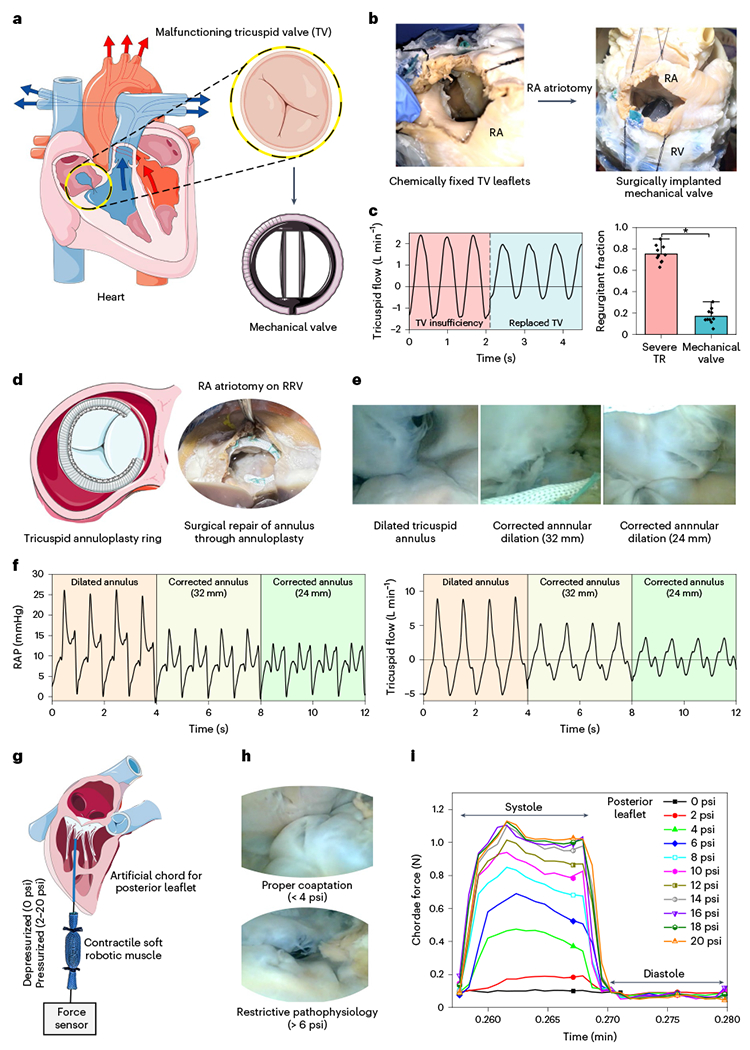
A platform for testing right heart interventions. **a**, As an example, the benchtop RRV model is used to test devices for TR. In this scenario, a mechanical valve is substituted for the damaged valve. **b**, To replace the native valve, a surgical right atrial atriotomy is performed. **c**, TR before and after intervention, shown by flow measurements for three cycles and a regurgitant fraction calculated over 10 cycles, demonstrating that the ability to study both healthy and pathological tricuspid flow in the same model allows for more accurate and comprehensive comparisons of the impact of different interventions on the cardiovascular system. Data show average ± s.d. for 10 cycles (*P < 0.0001 (exact value: 1.18631 × 10^−7^), calculated using one-way ANOVA (Tukey test with a significance level of 0.05)). **d**, Surgical implantation of prosthetic annuloplasty rings (32 mm and 24 mm) at the tricuspid annulus through right atrial atriotomy. **e**, Endoscopic view showcasing leaflet coaptation before and after the annulus correction. **f**, Hemodynamic data illustrating improved tricuspid flow and RAPs after annulus correction. **g**, Integration of a McKibben-based soft robotic muscle into the RRV to simulate papillary motion and to measure chordae force or tension. **h**, Tricuspid leaflet coaptation was visualized through an endoscopic camera after varying the input pressure (0–20 psi) on the posterior leaflet chordae. **i**, Measurement of force on the leaflet during systole while varying the input pressure from 0 psi to 20 psi.

In Fig. 8, we demonstrate the use of our RRV platform for repairing RV dysfunction caused by TR. The native TV leaflets were rendered dysfunctional through chemical fixation using formaldehyde. The malfunctioning leaflets were then surgically removed, and a 19-mm mechanical heart valve (St. Jude Medical) was surgically implanted at the tricuspid annulus (Fig. 8b). To assess the degree of regurgitation, we measured the tricuspid flow and observed a reduction in reverse flow compared to the pathological TV. The results showed a significant improvement in the tricuspid flow after the replacement of the mechanical valve, with a regurgitant fraction of 0.17 ± 0.07 compared to the severe TR fraction of 0.75 ± 0.08 (Fig. 8c).

### TV repair.

Dilation of the TV annulus can cause valvular dysfunction, leading to TR. TV repair aims to restore function by reshaping the annulus using a prosthetic annuloplasty ring. Selecting the right ring size is crucial to achieve proper annular reduction without under- or over-constriction. To test the hemodynamic consequences, we implanted different-sized rings (32 mm versus 24 mm) to assess the potential of RRV for investigating TV repair (Fig. 8d). An endoscopic camera was used to observe the leaflet coaptation both before and after the annulus correction (Fig. 8e and Supplementary Movie 5). Hemodynamic data indicated improved flow and pressures after annulus repair (Fig. 8f). The results showed a gradual reduction in RAP when comparing the dilated, unrepaired annulus with the 32-mm and 24-mm rings. TR decreased across both cases, with the most improvement seen with the downsized 24-mm ring. This study illustrates our model’s ability to represent the hemodynamic consequences of TV repair with a prosthetic annuloplasty ring in a clinically relevant context.

### Simulating chordae force and papillary muscle motion.

**Extended Data Fig. 6 |:**
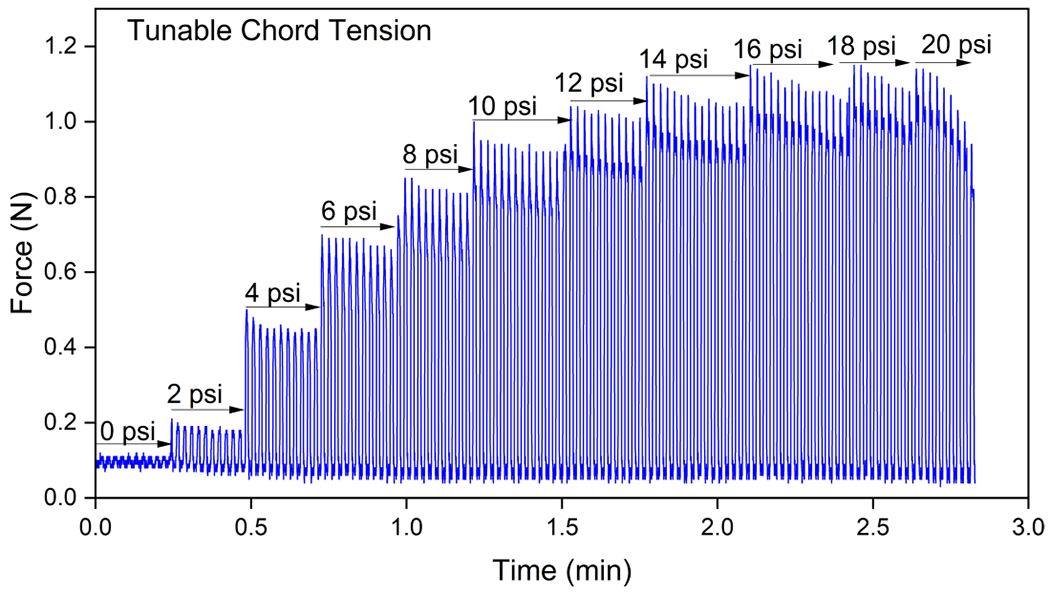
Recreating chordae tension. Measuring leaflet force during systole, with input pressure ranging from 0 to 20 psi, results in tunable force from 0.11 to 1.15 N to mimic clinical chordae tension.

In typical physiologic conditions, papillary muscles contract during systole, aiding valve function and preventing TV prolapse by applying tension to the chordae tendineae. In specific clinical situations, such as after right-sided myocardial infarction, patients may experience papillary muscle dysfunction or chordae tendineae rupture, resulting in TV malfunction and potential right ventricular failure. Soft robotic myocardium in RRV facilitates the study of clinically relevant pathophysiology involving chordae tendineae and papillary muscles. To simulate papillary muscle contraction during systole in the RRV model and to measure clinically relevant tension in the chordae, we integrated a McKibben-based soft robotic muscle into our robotic ventricle. The contractile muscle of length 65 mm was attached to the posterior leaflet’s chordae on one side and to a force sensor on the other (Fig. 8g and Supplementary Fig. 3). The pneumatic input pressure (ranging from 0 psi to 20 psi) applied to the muscle governs leaflet coaptation and dictates the resulting force or tension in the chordae. Figure 8h shows tricuspid leaflet coaptation as observed through an endoscopic camera after varying the input pressure on the posterior leaflet chordae. At higher input pressures (>6 psi), improper leaflet coaptation, mimicking restrictive pathophysiology in the RRV, is evident. The force measured on the leaflet during systole while varying the input pressure from 0 psi to 20 psi is tunable, spanning a range from 0.11 N to 1.15 N (Fig. 8i and Extended Data Fig. 6). Literature reports tricuspid chordae tension or papillary muscle forces in the range of 0.1 N to 0.4 N for healthy cases and 0.2 N to 0.7 N for diseased cases resulting from changes in the TV apparatus, such as a dilated annulus or papillary dysplasia^25^. This ability to recreate and adjust chordae tension using a contractile soft robotic muscle in RRV can replicate papillary muscle dysfunction in a laboratory setting.

## Discussion

Cardiac surgery has seen an uptick in the use of benchtop simulators for cardiovascular research, device testing and procedural demonstration^4,26^. Although simulators replicating left heart dysfunction have progressed, in vitro simulators accurately mimicking the hemodynamics, biomechanics and pathologies of the RV lack research^9,11,27^. This is a critical area of study because the RV is highly susceptible in many patients with repaired or palliated congenital heart disease and pulmonary hypertension^28,29^. RV-focused mechanical devices are currently being tested in animal studies, which are difficult to set up, expensive and often result in fatalities^8,30^. We aim to decrease the reliance on animal testing for evaluating the hemodynamic performance of intracardiac devices by replicating the associated pathophysiology of the RV on a laboratory bench using soft robotic techniques.

In the RRV, we recreate healthy and diseased right-sided hemodynamics by replicating the natural physiological function and mechanics of the RV. This involves designing a synthetic, soft robotic myocardium that imitates the sequential and peristaltic motion of the RV and its distinct fiber orientation. The pumping of fluid through contraction of the synthetic myocardium drives the valve motion during systole. The intact papillary muscles and chordae tendineae in the endocardial structure prevent the TV from prolapsing during ventricular contraction. The RRV simulator is a versatile tool compatible with various imaging modalities, such as echocardiography, CT and MRI, for studying anatomy and function. Existing MRI-compatible setups are limited in scope, mainly assessing LV hemodynamics with ex vivo pig hearts and a short lifespan^11^. Additionally, existing echocardiography tools are optimized for LV studies, making RV geometry and volume analysis challenging. Our beating benchtop model, with biomimetic motion, could improve RV imaging tools by measuring anteroposterior RV motion and identifying anatomical landmarks.

Apart from the interventions discussed, additional surgical and percutaneous methods for TV diseases include chord repair, edge-to-edge transcatheter repair and interventional catheter-based valve replacement. Transcatheter procedures, although complex, have gained popularity but have steep learning curves. RRV offers an effective solution for practicing these techniques and testing device efficacy in a simulated beating heart environment. It can also be adapted for training on cadaveric human hearts, providing an alternative to porcine hearts. This platform can also serve as a testbed for surgical procedures, including right heart catheterization for hemodynamic assessment^30^. The RRV model can support hands-on training and knowledge transfer, aiding in education and skill development. It can help diagnose and plan treatment for right heart disorders, such as pulmonary stenosis and congenital heart diseases^2,3,28,29^. Replicating hydraulic after-load and RV–PA coupling can enhance diagnostics and corresponding therapeutics. RV regional wall motion abnormalities are substantial in various conditions and can improve risk assessment clinically. The ability of the RRV to replicate both regional and global RV dysfunction can be useful for studying their impact on hemodynamics, facilitating evaluation of treatments and risk stratification^31–33^.

This study focused on demonstrating the recreation of right heart function as a proof of concept due to its crucial role in a multitude of pathologic clinical conditions. Future efforts can involve simulating the LV using similar strategies. By incorporating the functional left heart with the right heart circulation, a comprehensive cardiovascular simulator can be created. Such a simulator would hold great potential for research investigations in cardiac mechanics, flow dynamics and intervention testing including implantable intracardiac devices such as valve prostheses and left ventricular assist devices. Moreover, right heart diseases often coexist with left heart dysfunction. Therefore, simulating both sides of the heart within the biohybrid robotic in vitro simulator would enable detailed investigations into right heart pathology and facilitate intervention optimization. The primary focus of this work was to use the RRV as a research tool for testing intracardiac devices and recreating pathology, but it has the potential for future repurposing as a soft robotic total artificial heart. This can be achieved by optimizing the biocompatibility of the endocardial scaffold, possibly by replacing the current version with a biocompatible and hemocompatible tissue scaffold through a decellularization process. Alternatively, advancements in high-resolution, multi-material 3D printing could enable the reanimation of fully synthetic heart models using the soft robotic techniques outlined in this study.

Some limitations of this study are noted. The simulator has effectively replicated the symptoms of systolic dysfunction. However, it lacks the features of RV failure that are associated with dilation because the cavity size is restricted by the soft robotic myocardium, although this could be added in (along with adaptive control) in the future. The manufacturing process for the biorobotic hybrid heart is labor intensive and requires expertise. The fabrication process for synthetic myocardium involves manual casting and molding techniques. To overcome these limitations, a procedural manufacturing workflow can be adopted, incorporating diffusion tensor cardiac magnetic resonance (DT-CMR) and computer-aided design for accuracy. By using super-resolution DT-CMR images, we can identify the unique ventricular geometry and helical architecture specific to each case. Based on these findings, we can develop a 3D printable model of a silicone myocardial matrix. This model will incorporate channels or cylindrical voids for soft robotic actuators, faithfully replicating the observed helical and circumferential architecture. This approach would aim to create a robust and scalable heart model that surpasses the limitations of manual processing and is part of our future work. A specialized dissection technique was employed on a harvested porcine heart to remove non-functional myocardial tissue, requiring a comprehensive understanding of cardiac anatomy and myocardial fiber architecture to minimize defects. Despite efforts, minor defects may still occur due to human error. However, these issues can be addressed in the future with custom-designed techniques, such as automated tissue dissection. Although theoretically possible to create an automated tissue peeler for this purpose, it would necessitate expertise in robotics, automation and real-time control and feedback to achieve precise automation and design.

The RRV employs an ex vivo tissue scaffold to illustrate structural aspects, as no synthetic alternatives can currently replicate the wide range of material properties, including those ranging from 10 s of kPa to the sub-GPa range, with high resolution, including sub-feature sizes down to the sub-millimeter range. Although this approach enables high anatomical fidelity, it does not eliminate the need for animal testing. Precise anatomical representation is crucial for certain aspects, such as TV physiology and its interaction with adjacent structures, but it may not be as critical in other applications where valvular structures are less important and lack intricate interdependencies with surrounding elements. In such cases, fully synthetic models with soft robotic actuation may suffice. In the future, advancements in manufacturing techniques, such as multi-material high-resolution 3D printing, may eliminate the reliance on ex vivo tissue for accurate intracardiac structure representation. Such synthetic models would offer the advantage of representing geometric variations in heart anatomy, enabling the simulation of patient-specific anatomies with structural abnormalities or specific disease states. This study lacks comprehensive testing and evaluation of the durability and resilience of the RRV. Regarding the endocardial scaffold component, we observed that formalin-fixed and treated tissue can remain intact for several months to a year when kept hydrated and stored below 4 °C. In contrast, the soft robotic myocardium is expected to have a prolonged shelf life due to its chemical inertness. However, it may exhibit material hysteresis and eventual failure after a considerable number of cycles due to issues including delamination of the actuators, pinhole leaks in the silicone and coupling between the silicone and tissue. Although we conducted empirical testing for more than 10,000 actuation cycles during device testing and data collection, we have not performed long-term fatigue testing, which is a current limitation of this work. Furthermore, this study focuses on isolated RV dysfunction and does not consider RV–LV interactions, which can be explored in future research.

In conclusion, the RRV simulator, a biohybrid robotic right heart cardiac simulator, focuses on recapitulating the various pathologies of the RV in a tunable benchtop setting. It uses a myocardial substitute to recreate cardiovascular biomechanics and highlights the significance of changes in RV structure, function and loading on hemodynamics and clinical markers. With its high-fidelity simulation capabilities, this platform has the potential to aid in the development of intracardial interventions and tools for evaluating and treating RV dysfunction. In the future, it may serve to further advance understanding of the underlying pathophysiology of right heart failure.

## Methods

### Study design and overview

This work was carried out following Massachusetts Institute of Technology (MIT) Institutional Animal Care and Use Committee protocol number 0121-003-24 and adhering to the National Research Council’s guidelines for the ethical treatment of laboratory animals. Our study describes an anatomically and functionally accurate benchtop cardiac model, integrating organic components with synthetic soft robots to drive right-sided hemodynamic circulation. We used chemically treated ex vivo hearts with preserved mechanical properties and removed most of the thick myocardial tissue from the septal wall while retaining the endocardial lining and intracardiac components, such as the trabeculae, valves, papillary muscles and chordae tendineae. To ensure a conformal fit of the synthetic myocardium, we cast the outer geometry of the isolated RV with fast-curing silicone elastomer to create an outer mold and then place the robotic elements in desired locations. The orientation of robotic actuators in an elastomeric matrix is based on physiological tissue architecture, generating complex shortening and peristaltic motion. We developed a computational framework to optimize and predict the biomimetic motion and function of the soft robotic myocardium for generating right-sided ejection volumes and pressures. Finally, we connect the robotic heart to an instrumented flow loop to generate physiological SVs, pressures, waveform characteristics and related hemodynamic indexes. Our findings show substantial agreement with healthy in vivo pig data and reproduce classic hemodynamic patterns of RV dysfunction caused by abnormal loading conditions or contractile insufficiency, consistent with pathological data from human literature. The RRV simulator is compatible with multimodality imaging techniques, including echocardiography and MRI. It also allows for direct visualization of valve leaflet motion through the integration of an endoscopic camera and an optically clear blood analog. CT imaging enables visualization of the anatomy and structure of the robotic RV. Through proof of concept, we verified the ability of the robotic heart to reproduce complex movements of the RV free wall, septum and valves in both healthy and diseased conditions. RRV facilitates the approximation of non-contracting papillary muscles during RV wall contraction, bringing the chordae tendineae closer together for proper valve coaptation during systole. During diastole, the papillary muscles move farther away as the ventricular wall relaxes, allowing the TV to open. Existing literature supports the idea that papillary muscle approximation reduces systolic tethering forces on the chordae tendineae, improving valve function^34^. We validated the isolated RRV simulator in a healthy large animal model, reproducing the flow patterns within the RV as confirmed by 2D flow MRI. Additionally, we investigated the feasibility of surgically replacing a diseased TV with a mechanical heart valve and repairing a dilated tricuspid annulus using commercial annuloplasty rings in the model. The simulator has potential for testing and developing precise, targeted intracardiac interventions to treat RV heart failure. Dynamic organs, such as the heart, have intricate anatomical structures comprising composite materials with diverse mechanical properties. For evaluating the performance of implantable cardiac devices, simulating accurate anatomical structures, such as valves, papillary muscles and chordae tendineae, is crucial. Replicating these features and mechanical properties accurately through synthetic manufacturing methods presents considerable challenges. However, the hybrid approach used in RRV, combining explanted endocardial structures animated with soft robotics, effectively addresses these challenges.

### Manufacturing of the RRV

Before processing, explanted hearts were washed with 1× PBS to remove all blood and clots. Freshly excised porcine hearts were then preserved in a 10% neutral-buffered formalin (Sigma-Aldrich) for 18 h using gentle agitation, followed by thorough washing with 1 M PBS. The myocardial tissue from the LV and intraventricular septum were hand-dissected following the native fiber orientation^35^. The epicardial and myocardial tissues were manually dissected from the interventricular sulcus, starting at the front. Layer by layer, the LV free wall tissue was carefully removed until the fibers changed direction, differentiating the RVs and LVs. In the LV, dissection continued until the fibers became helical at 60°, marking the depth of the endocardial layer. The RV part of the interventricular septum was trimmed to protect the endocardial layers and intracardiac structures, removing myocardial tissues.

To restore the mechanical properties of the heart muscle and the pliability of the valves, the formalin-fixed endocardial scaffolds were treated with decellularization surfactants, consisting of 10% (w/v) sodium deoxycholate (Sigma-Aldrich) and 10% (w/v) Triton X-100 (Thermo Fisher Scientific) in 1× PBS for 1 week under gentle agitation at room temperature^36^. The decellularization solution was replaced and replenished three times during the weeklong process. Once the decellularization process was complete, the endocardial scaffold was thoroughly washed with distilled water under gentle agitation at room temperature for 24 h, with frequent solution changes to ensure the complete removal of residual detergent from the tissue.

To match the highly variable endocardial scaffold of each heart, a heart-specific soft robotic myocardium was developed. A silicone replica of the endocardial scaffold was created using a negative mold cast with Ecoflex 00-35 FAST (Smooth-On) after the myocardial dissection process. The dimensions of four circumferential actuators for the right ventricular free wall and three septal actuators were measured to fabricate the individual soft robotic elements, which are described in the next section. To align and position the actuators spatially, they were encased in a passive matrix layer of silicone with a thickness of approximately 6 mm. The silicone replica was used to create the appropriately sized silicone encapsulation. Once cured, the resulting structure, called the soft robotic myocardium, was attached to the endocardial tissue scaffold using a custom-made tissue/silicone adhesive^37^. The tissue/silicone adhesive is synthesized by combining a SYLGARD 184 Silicone Elastomer Kit with silane crosslinkers (Sigma-Aldrich) and a platinum(0)-1,3-divinyl-1,1,3,3-tetramethyl catalyst (Sigma-Aldrich).

### McKibben soft robotic actuators

The soft robotic actuators consist of three main components: a thermoplastic elastomer (TPE) bladder, a thermoplastic polyurethane tubing and a PET expandable braided mesh (Supplementary Fig. 1). The TPE bladder (Stretchlon 200) is sourced from Fibre Glast Developments Corporation. The 1/8-inch polyurethane tubing is obtained from McMaster-Carr, and the 1/4-inch PET mesh was purchased from TechFlex. These actuators are designed to fit different regions of the heart with varying bladder lengths and a 12-mm diameter. The bladder is shaped through thermal forming by heat-sealing two TPE layers on a 3D-printed mold at 300 °F for 4 s. The polyurethane tubing is then inserted and sealed with urethane adhesive (Ure-Bond II, Smooth-On), and the braided mesh is coated with Ecoflex 00-30 to prevent kinking. The bladder is connected to the expandable mesh, and the ends of the actuator are hand-sewn together using Kevlar thread. The actuators are quality tested by subjecting them to approximately 500 cycles of pressurization to 20 psi using a custom-made electropneumatic control system. This system uses electropneumatic pressure regulators and valves from SMC Pneumatics, enabling the customization of pressure waveforms and control over heart rate and systolic/diastolic phase ratios through the analog input^38^.

### Uniaxial tensile testing of myocardial tissue

Uniaxial tensile testing was conducted on myocardial tissue using rectangular strips measuring approximately 40 mm × 20 mm with a thickness of about 10 mm. These strips were obtained from porcine hearts and categorized into three groups: fresh tissue, myocardial tissue subjected to chemical fixation and muscle tissue treated with fixation followed by surfactant treatment. For each type (*n* = 3), the tissues were uniaxially stretched at a rate of 10 mm per minute using an Instron 5944 mechanical tester equipped with a 2-kN load cell. Although the tissue is viscoelastic, here we just calculated the elastic modulus in the linear elastic range according to Hooke’s law, based on the engineering stress versus strain plots.

### Micro-CT visualization

The structure and architecture of the RRV were analyzed through micro-CT using Skyscan 1276 X-ray microtomography from Bruker. During imaging, aluminum and copper filtering were used, and a rotation step of 0.647° was employed in step-and-shoot mode. The reconstructed data were visualized using DataViewer (Bruker).

### Optimizing soft robotic myocardium with computational model

We replicated the native RV myocardium fiber architecture using a two-layer design in our soft robotic myocardium model. This model consisted of linearly contracting active elements arranged either circumferentially or longitudinally in a silicone matrix along the RV wall and septum. Each layer had three actuators in each direction to assess RV motion and hemodynamics during systole. We chose a spatial density of three discrete actuators equidistantly placed in each layer to cover the entire length of the RV wall obtained from 30–40-kg swine models. For larger hearts, the same two-layer design could be used with more actuator units. We imposed orthotropic strain to simulate structural deformation, and an additional actuator was added to the base of the RV free wall to mimic the contractile effect of the basal region.

The idealized RV and actuators were created using SolidWorks 2022 (Dassault Systèmes) and imported into Abaqus 2022 (Simulia, Dassault Systèmes) for nonlinear explicit dynamic analysis. The anisotropic strain was applied to the actuators to replicate local deformation following orthotropic expansion via thermal analogy^39^. A 10-node C3D10M tetrahedral element simulated the passive soft matrix as a neo-Hookean hyperelastic material with parameters C10 = 0.0113333 and D1 = 1.96. Additionally, the circumferential and longitudinal actuators, designed to provide flexibility in contracting, were represented in the model as linear elastic materials with a Young’s modulus of 15.34 MPa. Intraventricular hemodynamics were simulated using a fully coupled fluid–structure interaction (FSI) technique. Large eddy simulation (LES) was used in XFlow 2022x to model flow patterns, and the immersed boundary method (IBM) avoided mesh issues.

To simulate the intraventricular hemodynamic characteristics of the RV, a fully coupled FSI modeling technique was employed. Within this approach, an LES turbulence model was used in XFlow 2022x by Dassault Systèmes to replicate flow patterns within the RV. To facilitate grid generation and avoid complications related to mesh movement and regeneration caused by deformations, the IBM was applied. Traditional computational fluid dynamic models commonly rely on Navier–Stokes solvers. However, these methods have notable limitations, including the need for complex re-meshing and the use of overly empirical turbulence simulations, such as Reynolds-averaged Navier–Stokes (RANS)^40,41^. To overcome these limitations, the IBM approach was adopted. This allowed for a two-way coupling computation between the structural and fluid flow domains. The blood was represented as an incompressible Newtonian fluid with a density of 1,050 kg m^−3^ and a dynamic viscosity of 0.0035 Pa·s. In the initial stage of the simulation, the RV cavity was filled with lattice particles, using a grid resolution of 0.6 mm (as shown in Fig. 2b). Grid independence was assessed by using three different grid sizes (lattice resolutions of 1.0 mm, 0.8 mm and 0.6 mm). Convergence of the stability parameter was attained, with a difference of 0.92% between the medium-quality grid and the fine grid. Consequently, the medium grid with a 0.6-mm resolution (comprising approximately 205,000 elements) was grid independent.

The study also involved an assessment of transient flow dynamics within the RRV and its interaction with the wall deformation caused by the actuators. This was achieved by defining the RV wall as a moving boundary condition between the structural and fluid flow domains, using a timestep of 1 × 10^−5^ s. To simulate an outflow boundary condition between the RV and the PA, an outlet surface was created at the interface plane. This outlet surface was provided with a time-dependent physiological PAP waveform. A time-dependent pressure pattern (with phases of 30/15 mmHg) representing the normal physiological pressure waveform in the PA was assigned to an artificial cap created at the basal plane. This cap acted as an outflow boundary condition between the RV and the PA. It is worth noting that the diastolic filling stage was not captured, as the outflow surface was not defined to allow for backflow. The simulation began with the RV in a resting state, and then a hydraulic actuation ramp was applied to the actuators for 300 ms, mimicking the contraction during the systolic phase. Each FSI simulation took approximately 22.5 h to complete and was performed on a desktop PC equipped with a 3.0-GHz i7-9700 processor, featuring eight cores and 32 GB of RAM. Throughout the simulation, velocity and pressure field distributions were recorded at a frequency of 10 Hz. Additionally, the ejection volume was calculated by integrating the outflow at the outlet boundary over time.

### Mock circulatory flow loop

A mock circulatory loop comprising hydraulic and mechanical components was employed to simulate the right heart circulation. The loop featured in-house-built acrylic compliance chambers that represented venous and pulmonary compliances as well as on–off ball valves (McMaster-Carr, 4796K71) that simulated pulmonary arterial and pulmonic vascular resistance. The RRV was connected to the loop and actuated at 60 bpm using a custom-made electropneumatic control box. The actuation, resistance and compliance levels were adjusted to obtain the desired right-sided hemodynamics. A blood mimic fluid composed of 40 v/v % propylene glycol in deionized water with a dynamic viscosity of 4.3 ± 0.8 mPa·s was used, and hemodynamic parameters were measured with pressure sensors (PRESS-S-000, PendoTECH) and recorded with PowerLab (ADInstruments) and LabChart Pro version 8.1.16 software (ADInstruments). The pressure sensors were positioned at the RA, RV and PA via 3.5-F umbilical vessel catheters (Cardinal Health) to measure the biphasic pressure waveforms. The ultrasonic flow probe (ME 13 PXN, Transonic), connected to a T420 multichannel research console (Transonic Systems), was mounted directly onto the PA to record the outflow downstream to the valve. A 1,080-P HD endoscopic camera from NIDAGE was used to capture videos at 30 frames per second (fps) to visualize the valve motion.

### Animal handling and surgical procedures on porcine models

Animal experiments were carried out following MIT Institutional Animal Care and Use Committee protocol number 0121-003-24 and adhering to the National Research Council’s guidelines for the ethical treatment of laboratory animals. Three female Yorkshire swine (sourced from the Cummings School of Veterinary Medicine at Tufts University) weighing between 30 kg and 40 kg (age 3 months) were subjected to intubation, placed on mechanical ventilation using an Aestiva 5 device from Datex-Ohmeda and administered general anesthesia, which was maintained at a level of 2–3% isoflurane. Arterial and venous femoral lines were inserted to monitor systemic arterial pressure and deliver medications, respectively. A median sternotomy was performed to access the chest, and 5-F catheters were carefully introduced into the RA, RA and PA. These catheters were secured in place with purse-string sutures. Flow probes were also positioned around the PA and IVC after dissection of the aortopulmonary window. All pressure and flow probe lines were passed through a subxiphoid skin incision. The sternum was then closed using sternal wires, and the subcutaneous layers and skin were sutured in layers to ensure proper chest closure. Subsequently, the animals were humanely euthanized with pentobarbital at a dose of 100 mg per kilogram of body weight.

### In vivo hemodynamics acquisition

To record the pressure (PRESS-S-000, PendoTECH) and flow (ME 13 PXN, Transonic Systems) sensor data, a high-performance data acquisition system from ADInstruments, specifically the PowerLab 35 series, was used with a 1-kHz sampling frequency. The data were monitored in real time during the in vivo study using LabChart Pro version 8.1.16 software (ADInstruments). To process the pressure and flow data recordings and remove high-frequency noise, we used a 10-Hz low-pass digital filter in LabChart.

### In vitro hemodynamics validation

In vivo hemodynamics were measured through catheterization and implanted flow probes and recorded using a high-resolution data acquisition system (Fig. 3a,b). Porcine hearts were then harvested and converted into robotic hearts, which were connected to a mock circulatory loop. The loop was instrumented with ultrasonic flow sensors, pressure sensors, compliance chambers and resistive elements to record and tune the real-time hemodynamic data (Fig. 3c). The heart was actuated at 87 bpm using a custom electropneumatic control box, enabling user adjustment of input parameters, such as systolic-to-diastolic duration ratio. An endoscopic camera in the circuit enabled visualization of intracardiac structures.

### Echocardiography

Heart and valvular motion were assessed using echocardiography with a 2D B-mode. The Philips Epiq CVx cardiovascular ultrasound system with XL14-3 and X5-1 transducers was used. To obtain a precise view of the RV wall and valve motion, the transducer was placed directly onto the biohybrid robotic heart for epicardial imaging on the benchtop. Live animal images were obtained with epicardial 2D imaging. The data were analyzed and visualized using Q-Vue 2.2 (Philips).

### Determining clinical parameters

The equations provided below were employed to estimate the clinical metrics used for assessing RV function.

1. Pulmonary transvalvular pressure gradient (PVPG) = pressure difference across the pulmonary valve = *Peak RVSP* – *peak PASP*
2. Pulmonary regurgitation $\left(PR\right)=\frac{backward\;flow\;volume}{forward\;flow\;volume}$
3. Pulmonary artery compliance (PAC) = (*SV*)/(*PASP – PADP*)
4. Right ventricular stroke work index $index=\frac{SV}{BSA}x\left(mPAP-RAP\right)$
5. Body surface area (BSA) = 0.0734 × body weight^0.656^

PCWP, pulmonary capillary wedge pressure.

### 2D flow MRI

To enable imaging in the MR environment, all ferromagnetic components in the mock circulatory loop were replaced with plastic parts. The control system for the cyclic actuation of the robotic heart was positioned outside the MR room. Imaging was conducted using a 3-T clinical MRI system (MAGNETOM Skyra scanner, Siemens Healthineers). The voxel resolution for the 2D flow was 0.9464 × 0.9464 × 8 mm, and a simulated electrocardiogram signal with an RR period similar to the beating of the robotic heart was employed. The 2D flow was acquired with three-directional velocity encoding along the through-plane direction. Imaging parameters for the 2D flow MRI sequence were as follows: repetition time = 37.2 ms, echo time = 2.74 ms, flip angle = 7°, velocity encoding (VENC) sequence =50 cm s^−1^ to 150 cm s^−1^, temporal resolution = 25 frames per cycle and 2D matrix size = 112 × 112. The analysis of MRI phase-contrast 2D flow data was performed using MATLAB 2020a (MathWorks). The quality of the data used to generate velocity maps may be impaired by sources that induce phase offsets, such as eddy currents, in a 2D flow MRI signal. The data processing included noise masking, velocity anti-aliasing and corrections for eddy currents. Masking was created from the magnitude of raw data in the area where the structure of the biorobotic hybrid heart was evident. Construction of velocity vectors from the 3D VENC dataset (v_enc_, 12-bit) was accomplished by using the phase data (d_X_, d_Y_ and d_Z_) indicating velocity in the in-plane directions (*x* and *y*) and out-of-plane direction (*z*) as shown below: (6) $v_{i}=\left(\frac{d_{i}}{4096}\right)*v_{enc}$

i=[x,y,z]

The created mask was used to overlay the phase data at the segmented borders, allowing for better flow visualization and quantification. The phase data were converted into the point cloud using the Visualization Tool Kit (VTK) libraries (VTK, Kitware). The point cloud was then loaded into a 3D flow visualization package, ParaView 5.9.1 (Sandia National Labs, Kitware). Color-coded velocity vectors from the masked area were used to visualize intraventricular blood flow patterns.

### TV annuloplasty

The native annulus was dilated by inserting an oversized balloon during chemical fixation, leading to annulus enlargement. We surgically implanted prosthetic annuloplasty rings (Edwards Physio Tricuspid, model 6200) at the tricuspid annulus through right atrial atriotomy (Fig. 8d). To assess the impact of ring size (32 mm versus 24 mm), we measured tricuspid flow and RAPs, using unrepaired dilated annulus as a control within the same heart.

### Reporting summary

Further information on research design is available in the Nature Portfolio Reporting Summary linked to this article.

## Extended Data

**Extended Data Table 1 |:** Unique features of RRV

	*Ex vivo* passive heart models	Perfusion Heart models (Langendorff and working heart)	Fully synthetic soft robotic heart models and synthetic pulse duplicators	RRV model
**Actuation**	Internal fluid pressurization through external pumps	Myocardial contraction through circulating the perfusate	• External pneumatic actuation in soft robotic models • External pulsatile pump in pulse duplicators	Controlled external robotic actuation
**Flow pattern**	Artificial	• No flow of fluid until Langendorff perfusion is ceased and normal sinus rhythm is established • Working heart model supports the natural flow	• Natural in soft robotic models • Unnatural in pulse duplicators due to rigid structures	Natural
**Pressure-volume relations**	Paradoxical	Physiological	• Physiological in soft robotic models • Paradoxical in pulse duplicators	Physiological
**Ventricular motion**	Unnatural expansion during systole	Biomimetic	• Biomimetic in soft robotic models • No wall motion in pulse duplicators due to rigid chambers	Biomimetic
**Valve motion**	Unnatural • Papillary muscles move away during systole • Higher tension in chordae during systole • Disrupted AV valve physiology	• Retrograde aortic flow in Langendorff model prevents aortic valve from opening • Competent AV valves, function declines over time	• Employ artificial bioprosthetic or mechanical heart valves • Existing soft robotic models lack AV valve apparatus • Pulse duplicators use isolated ex vivo AV valves, lack kinematic representation	Physiological • Papillary muscles approximation • Correct AV valve physiology • Papillary muscles contraction during systole to ensure chordae tension and prevent TR
**Shelf-life**	Up to a day	4 hours	Years	Months
**Scale**	Large animal models	Mainly employed in rat, guinea pig, and juvenile rabbit heart models, but adaptable to larger animals and human hearts with added complexity	Human-scale models, patient-specific models possible	Designed for large animal and human physiology
**Testing Setup**	Complex	Complex and laborious, requires pacing for stabilizing heart rhythm; cannot be used if the time between excision and perfusion is > 120 s, numerous arrythmias	Rapid and Robust	Sophisticated, requires skills and specialized equipment
**Function**	Recreates physiological HR, flow rate, and pressures	HR: Mainly bradycardic; PP≈ 10 mmHg; LVP: decline in function 5-10% per hour	Capable of recreating physiological HR, flow rate, and pressures	Fine-tunable setup that faithfully reproduces physiological HR, flow, and pressures
**Cardiac Anatomy**	Intact and accurate Intracardiac anatomy	Intact and accurate intracardiac anatomy	Simplified geometry or partial representation of the cardiac anatomy	Intact and accurate intracardiac anatomy

Comprehensive comparison highlighting the distinctive features and advantages of the hybrid soft robotic platform RRV in contrast to existing models. AV, atrioventricular; HR, heart rate; LVP, left ventricular pressure; PP, pulse pressure.

## Supplementary Material

Supplemental Video 5

Supplemental Video 4

Supplemental Video 3

Supplemental Video 2

Supplemental Video 1

Supplemental Information

## Data Availability

The data are available in the main article and Supplementary Information. Source data are provided with this paper. All additional data can be requested from the corresponding author.
