# Supplementary material for: Robotic right ventricle is a biohybrid platform that simulates right ventricular function in (patho)physiological conditions and intervention
Source: Nat Cardiovasc Res. 2023 Dec 8;2(12):1310–26. doi: 10.1038/s44161-023-00387-8 (PMC11343235; doi:10.1038/s44161-023-00387-8)
Supplement: Supplementary file 2 [file 44161_2023_387_MOESM2_ESM.pdf]

nature portfolio

Corresponding author(s): Ellen T. Roche

Last updated by author(s): Oct 28, 2023

Reporting Summary

Nature Portfolio wishes to improve the reproducibility of the work that we publish. This form provides structure for consistency and transparency in reporting. For further information on Nature Portfolio policies, see our Editorial Policies and the Editorial Policy Checklist.

Statistics

For all statistical analyses, confirm that the following items are present in the figure legend, table legend, main text, or Methods section.

n/a	Confirmed	
☐	☒	The exact sample size (*n*) for each experimental group/condition, given as a discrete number and unit of measurement
☐	☒	A statement on whether measurements were taken from distinct samples or whether the same sample was measured repeatedly
☐	☒	The statistical test(s) used AND whether they are one- or two-sided<br>*Only common tests should be described solely by name; describe more complex techniques in the Methods section.*
☒	☐	A description of all covariates tested
☒	☐	A description of any assumptions or corrections, such as tests of normality and adjustment for multiple comparisons
☐	☒	A full description of the statistical parameters including central tendency (e.g. means) or other basic estimates (e.g. regression coefficient) AND variation (e.g. standard deviation) or associated estimates of uncertainty (e.g. confidence intervals)
☐	☒	For null hypothesis testing, the test statistic (e.g. *F*, *t*, *r*) with confidence intervals, effect sizes, degrees of freedom and *P* value noted<br>*Give P values as exact values whenever suitable.*
☒	☐	For Bayesian analysis, information on the choice of priors and Markov chain Monte Carlo settings
☒	☐	For hierarchical and complex designs, identification of the appropriate level for tests and full reporting of outcomes
☒	☐	Estimates of effect sizes (e.g. Cohen's *d*, Pearson's *r*), indicating how they were calculated

Our web collection on statistics for biologists contains articles on many of the points above.

Software and code

Policy information about availability of computer code

Data collection	Skyscan 1276 X-ray microtomography from Bruker for micro CT data; computer-aided design (CAD) software SolidWorks 2022 (Dassault Systèmes) and imported into Abaqus 2022 software (Simulia, Dassault Systèmes) for in silico work; pressure sensors (PRESS-S-000, PendoTECH) and recorded with PowerLab 35 series (AD Instruments) and LabChart Pro v8.1.16 software (AD Instruments), ultrasonic flow probe (ME 13 PXN, Transonic), connected to a T420 multichannel research console (Transonic Systems Inc.) for hemodynamic data collection; The Philips Epiq CVx cardiovascular ultrasound system with XL14-3 and X5-1 transducers for echocardipgraphy; MAGNETOM Skyra scanner, Siemens Healthineers for flow MRI; A 1080P HD endoscopic camera from NIDAGE was used to capture 30 fps videos for visualizing valve motion.
Data analysis	The following data analysis, visualization, and plotting tools were used: Matlab 2020a (The Mathworks, Boston, MA); Visualization Tool Kit (VTK) libraries (VTK, Kitware, Inc., NY). The point cloud was then loaded into a 3D flow visualization package, ParaView 5.9.1 (Sandia National Labs, Kitware Inc., Los Alamos National Labs); CFD model data were generated with XFlow 2022x (Dassault Systemes).The reconstructed CT data was visualized using Dataviewer v1.5.6.2 64-bit (Bruker).The electrocardiography data was analyzed and visualized using Q-Vue 2.2 (Philips).The illustrations are made using BioRender.com and Adobe Illustrator 2023 and the data are analyzed and plotted using OriginPro 2021b software. Parts of some figures are directly adapted or modified using pictures from Servier Medical Art. Servier Medical Art by Servier is licensed under a Creative Commons Attribution 3.0 Unported License (https://creativecommons.org/licenses/by/3.0/).

For manuscripts utilizing custom algorithms or software that are central to the research but not yet described in published literature, software must be made available to editors and reviewers. We strongly encourage code deposition in a community repository (e.g. GitHub). See the Nature Portfolio guidelines for submitting code & software for further information.

Data

Policy information about availability of data

All manuscripts must include a data availability statement. This statement should provide the following information, where applicable:

- Accession codes, unique identifiers, or web links for publicly available datasets
- A description of any restrictions on data availability
- For clinical datasets or third party data, please ensure that the statement adheres to our policy

The data is available in the main article and supplementary information, and source data for the figures are included in this paper. All additional data can be requested from the corresponding author.

Research involving human participants, their data, or biological material

Policy information about studies with human participants or human data. See also policy information about sex, gender (identity/presentation), and sexual orientation and race, ethnicity and racism.

Reporting on sex and gender	No Research involving human participants is presented
Reporting on race, ethnicity, or other socially relevant groupings	Not applicable
Population characteristics	Not applicable
Recruitment	Not applicable
Ethics oversight	Not applicable

Note that full information on the approval of the study protocol must also be provided in the manuscript.

Field-specific reporting

Please select the one below that is the best fit for your research. If you are not sure, read the appropriate sections before making your selection.

☒ Life sciences ☐ Behavioural & social sciences ☐ Ecological, evolutionary & environmental sciences

For a reference copy of the document with all sections, see nature.com/documents/nr-reporting-summary-flat.pdf

Life sciences study design

All studies must disclose on these points even when the disclosure is negative.

Sample size	In vivo validation and benchtop testing of healthy physiology involved three animals and their respective explanted hearts. For disease modeling and clinical translation applications, a single heart per case was utilized due to the reproducibility of the soft robotic techniques and morphological variability on each endocardial scaffold. Each heart yielded a substantial volume of hemodynamic data, with each cardiac cycle contributing a data point for the analysis presented. Preliminary investigations, in the form of pilot studies, were conducted to assess the success rate of in vivo hemodynamics recording, with a focus on procedures free from complications or with minimal complications. Additional pilot studies were undertaken to determine the success rate of converting an explanted heart into a biohybrid robotic heart and effectively recreating the hemodynamics on the bench. All attempts were successful, and data from all three hearts are presented. The information gathered from these pilot studies was then used to ascertain the optimal sample size required for a robust study capable of establishing statistical significance among different groups.
Data exclusions	No data was excluded from analysis
Replication	Whenever possible, we employed N=3 replicates in our experiments. In the case of in vivo validation and benchtop testing, we utilized three animals. As for clinical applications on benchtop, we present proof of concept data for each type, each based on a single heart. All attempts (in vivo and benchtop) were successful, and data from all three hearts are presented.
Randomization	Robotic hearts for benchtop testing were randomly allocated to recapitulate pathological conditions. In the case of in-vivo hemodynamics, all swine underwent identical procedures and testing, eliminating the need for randomization.
Blinding	In in-vivo surgeries, animal recruitments, and data collection were carried out by a skilled veterinary and clinical team. However, for in-vitro or benchtop experiments, investigators were not blinded and had the flexibility to adjust parameters within the mock circulatory flow loop to replicate clinically relevant outcomes and assessments. This flexibility was necessary because the data from each explanted heart had to be combined with subject-specific in-vivo data from each animal for validation studies. In the case of in-vivo testing and data collection, all animals underwent the same procedure, making blinding unnecessary.

Reporting for specific materials, systems and methods

We require information from authors about some types of materials, experimental systems and methods used in many studies. Here, indicate whether each material, system or method listed is relevant to your study. If you are not sure if a list item applies to your research, read the appropriate section before selecting a response.

Materials & experimental systems

n/a	Involved in the study
☒	☐ Antibodies
☒	☐ Eukaryotic cell lines
☒	☐ Palaeontology and archaeology
☐	☒ Animals and other organisms
☒	☐ Clinical data
☒	☐ Dual use research of concern
☒	☐ Plants

Methods

n/a	Involved in the study
☒	☐ ChIP-seq
☒	☐ Flow cytometry
☒	☐ MRI-based neuroimaging

Animals and other research organisms

Policy information about studies involving animals; ARRIVE guidelines recommended for reporting animal research, and Sex and Gender in Research

Laboratory animals	Female Yorkshire swine (30-40kg), age 3 months, sourced from Cummings School of Veterinary Medicine at Tufts University, Grafton, MA
Wild animals	No wild animals were used.
Reporting on sex	Female Yorkshire swine were the choice of our animal model because of their availability, similarities to humans (Size and Anatomy), and ease of handling, as reported in literature.
Field-collected samples	No field collected samples were used in the study.
Ethics oversight	Animal studies were conducted in accordance with MIT Institutional Animal Care and Use Committee protocol #0121-003-24, following the National Research Council's Guide for the Care and Use of Laboratory Animals.

Note that full information on the approval of the study protocol must also be provided in the manuscript.
