# Supplementary material for: Robotic right ventricle is a biohybrid platform that simulates right ventricular function in (patho)physiological conditions and intervention
Source: Nat Cardiovasc Res. Author manuscript; Available in PMC 2024 Aug 23. (PMC11343235; doi:10.1038/s44161-023-00387-8)
Supplement: Supplemental Information [file NIHMS1970594-supplement-Supplemental_Information.pdf]

nature cardiovascular research

Technical Report https://doi.org/10.1038/s44161-023-00387-8

Robotic right ventricle is a biohybrid platform that simulates right ventricular function in (patho)physiological conditions and intervention

In the format provided by the authors and unedited

Supplementary Information

Table of contents:

Supplementary Figures 1-3

Descriptive captions of Supplementary Movies 1-5

Source data for the main figures (Figs 2c-2f, 3d-3g, 4a-4b, 7a-7c, 8c, 8f, and 8i) and extended data (Figs 1, 4-6) are available in separate .xlsx files

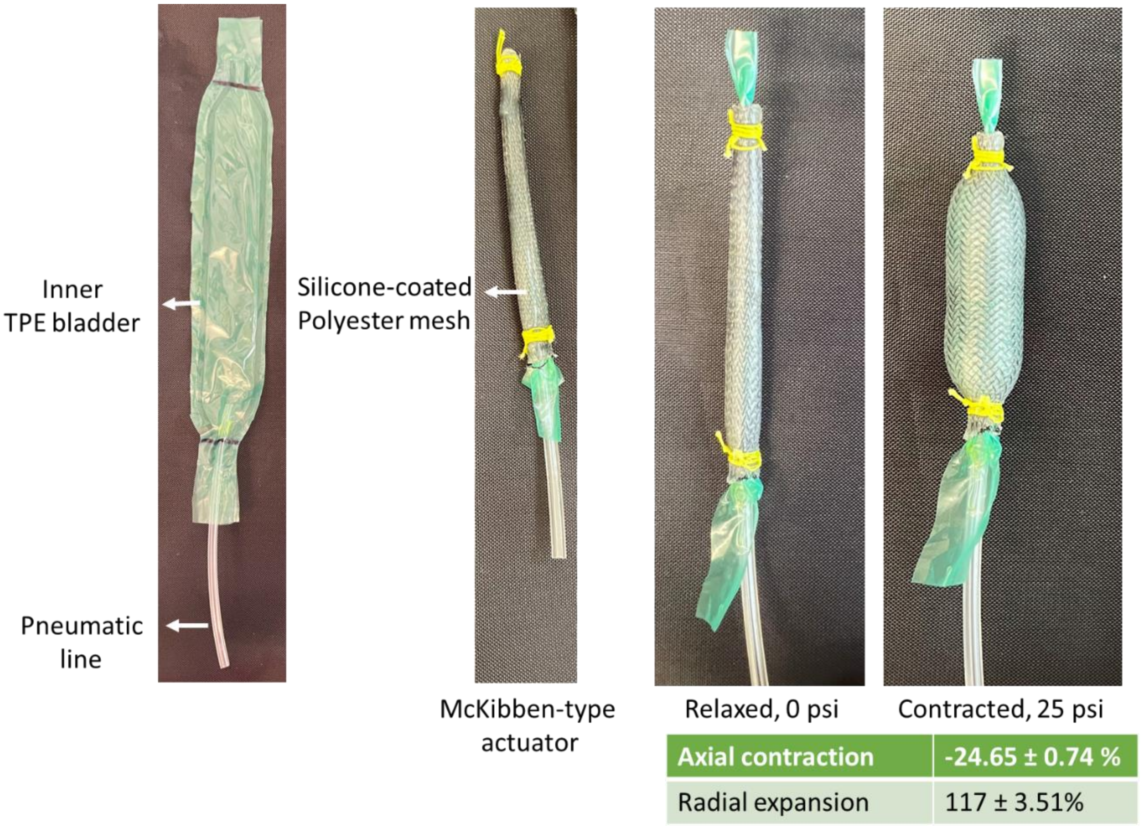

Supplementary Figure 1: Fabrication and characterization of McKibben-type soft robotic actuators. TPE, thermoplastic elastomer.

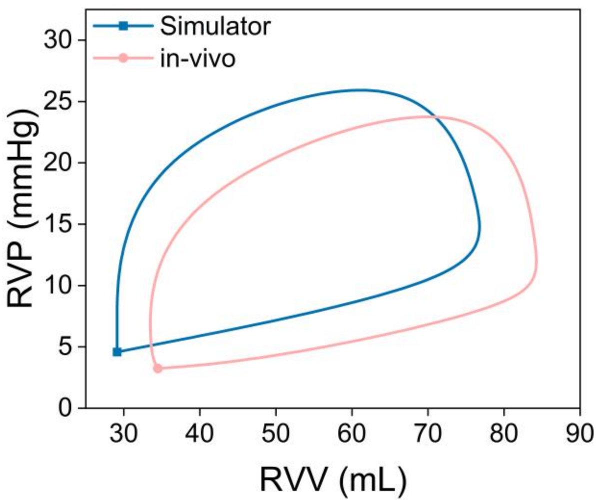

Supplementary Figure 2: The representative pressure-volume loops of the robotic right ventricle (RRV) simulator vs. an *in vivo* porcine model over one cardiac cycle.

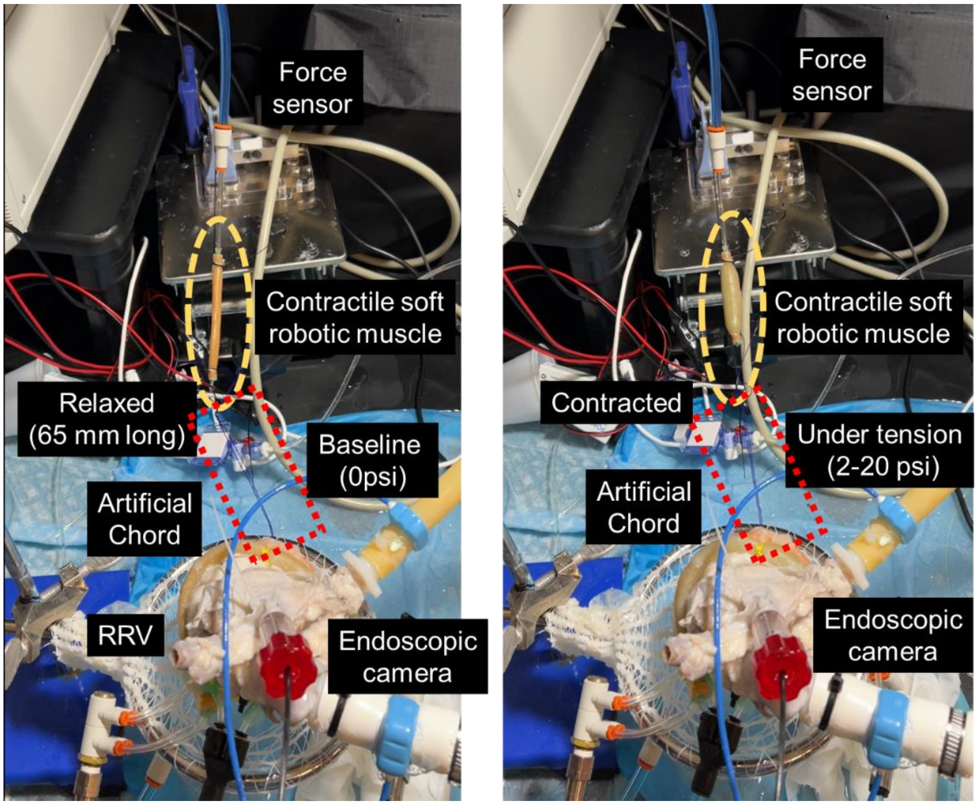

Supplementary Figure 3: Integrating a 65 mm McKibben soft robotic muscle into the robotic right ventricle (RRV), connecting it to the posterior leaflet chordae on one end and a force sensor on the other for recreating papillary muscle contraction on bench.

Supplementary Movie 1: Structural motion and flow profile of the RRV in a computational model. RRV, robotic right ventricle.

Supplementary Movie 2: Beating RRV in the *in vitro* mock circulatory flow loop recreating the right ventricular (RV) wall motion on a bench. RRV, robotic right ventricle.

Supplementary Movie 3: The wall motion and valve function of the RRV using magnetic resonance imaging (MRI), echocardiography, and endoscopic imaging. RRV, robotic right ventricle.

Supplementary Movie 4: Simulating various disease conditions related to right ventricular (RV) volume overload, systolic failure, and pressure overload, such as tricuspid regurgitation, myocardial infarction, and pulmonary artery hypertension.

Supplementary Movie 5: The surgical implantation of prosthetic annuloplasty rings at the tricuspid annulus effectively establishes RRV as a suitable platform for testing right heart interventions and evaluating their hemodynamic consequences. RRV, robotic right ventricle.
